# Spin–phonon coupling and magnetic relaxation in single-molecule magnets†

**DOI:** 10.1039/d2cs00705c

**Published:** 2023-06-28

**Authors:** Jon G. C. Kragskow, Andrea Mattioni, Jakob K. Staab, Daniel Reta, Jonathan M. Skelton, Nicholas F. Chilton

**Affiliations:** a Department of Chemistry, The University of Manchester Manchester M13 9PL UK jonathan.skelton@manchester.ac.uk nicholas.chilton@manchester.ac.uk; b Faculty of Chemistry, The University of the Basque Country UPV/EHU Donostia 20018 Spain; c Donostia International Physics Center (DIPC) Donostia 20018 Spain; d IKERBASQUE, Basque Foundation for Science Bilbao 48013 Spain

## Abstract

Electron–phonon coupling is important in many physical phenomena, *e.g.* photosynthesis, catalysis and quantum information processing, but its impacts are difficult to grasp on the microscopic level. One area attracting wide interest is that of single-molecule magnets, which is motivated by searching for the ultimate limit in the miniaturisation of binary data storage media. The utility of a molecule to store magnetic information is quantified by the timescale of its magnetic reversal processes, also known as magnetic relaxation, which is limited by spin–phonon coupling. Several recent accomplishments of synthetic organometallic chemistry have led to the observation of molecular magnetic memory effects at temperatures above that of liquid nitrogen. These discoveries have highlighted how far chemical design strategies for maximising magnetic anisotropy have come, but have also highlighted the need to characterise the complex interplay between phonons and molecular spin states. The crucial step is to make a link between magnetic relaxation and chemical motifs, and so be able to produce design criteria to extend molecular magnetic memory. The basic physics associated with spin–phonon coupling and magnetic relaxation was outlined in the early 20th century using perturbation theory, and has more recently been recast in the form of a general open quantum systems formalism and tackled with different levels of approximations. It is the purpose of this Tutorial Review to introduce the topics of phonons, molecular spin–phonon coupling, and magnetic relaxation, and to outline the relevant theories in connection with both the traditional perturbative texts and the more modern open quantum systems methods.

## Abstract 2

Electron–phonon coupling underlies many physical phenomena, but its microscopic origins are nuanced. This Review derives the spin–phonon interactions in molecules from first principles, and describes an implementation for molecular spin dynamics calculations.

## Graphical abstract

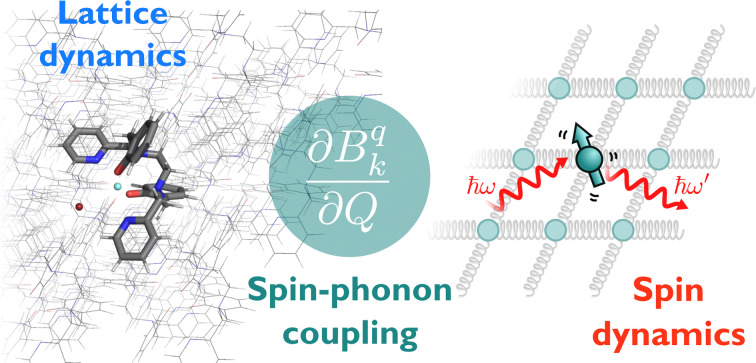

**Key learning points**

1. The nature of phonons in molecular crystals.

2. How spin–phonon coupling arises in molecules.

3. How the spin–phonon interaction drives magnetic relaxation.

4. How molecular spin dynamics and magnetic relaxation can be modelled with different levels of approximation.

5. How to perform first-principles calculation of the spin–phonon coupling.

## 1 Introduction

For the last thirty years, many inorganic, physical and computational chemists, along with experimental and theoretical physicists, have been captivated by the discovery,^1,2^ study and development of single-molecule magnets (SMMs). SMMs are molecules that possess a doubly-degenerate electronic ground state, the components of which have large uniaxial magnetic moments in opposing directions that can represent binary 1 and 0.^3,4^ The other low-lying excited electronic states generally have their magnetic moments oriented more towards the equatorial plane (*i.e.* perpendicular to the axis defined by the ground doublet); this energy difference between different orientations of the magnetic moment is the origin of magnetic anisotropy. The best-performing SMMs to-date are [DyCp^Me_5_^Cp^^i^Pr_5_^][B(C_6_F_5_)_4_] and [Cp^^i^Pr_5_^DyI_3_DyCp^^i^Pr_5_^] (Cp^Me_5_^ = C_5_(CH_3_)_5_ and Cp^^i^Pr_5_^ = C_5_(CH(CH_3_)_2_)_5_), which both show open magnetic hysteresis up to 80 K;^5,6^ the latter features strong magnetic interactions between the metal ions, which offers a new route to achieving improved performance.^7^

**Fig. 1:**
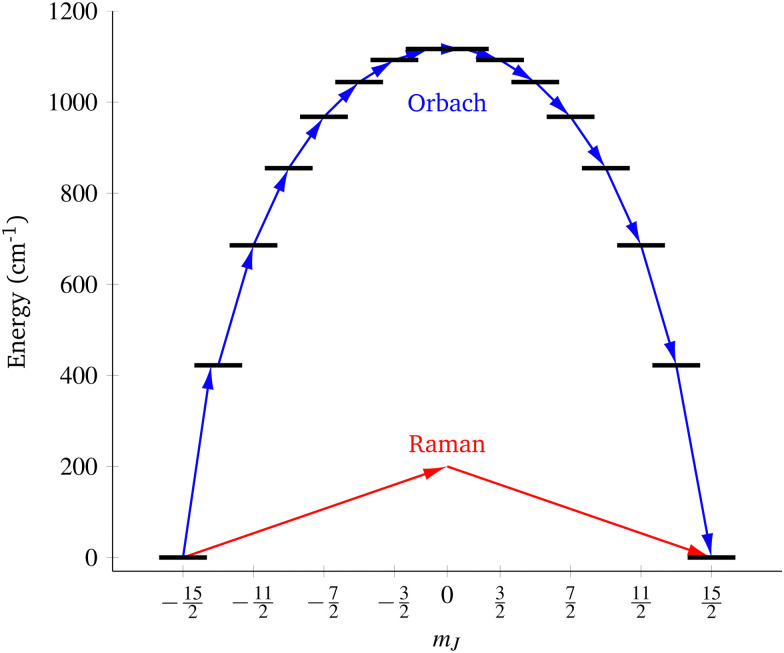
Two main mechanisms of magnetic relaxation arising from spin–phonon coupling in single-molecule magnets, Orbach (blue) and Raman (red), illustrated for the *J* = 15/2 multiplet of a Dy^3+^ SMM with a strong uniaxial crystal field.

There have been numerous recent reviews on SMMs,^7–10^ and it is not the purpose of this Tutorial Review to discuss the requisite ingredients for their design; indeed, this Review assumes a basic understanding of such matters.^4,11^ Rather, here we focus on the theory and calculation of magnetisation dynamics in SMMs as arising from spin–phonon coupling. While there are many contemporary theoretical works in this area,^12–18^ and some excellent reviews of the traditional methods,^19^ there exists a gulf between the modern parlance of these physics-based texts and the original works in the early to mid 20^th^ century to which the field constantly reflects.^20–23^ Hence, this work intends to contextualise modern molecular spin–phonon coupling theory with a backdrop of the original works, and to provide an explanation suitable for newcomers to the field – including the basics of lattice dynamics. Herein we focus on discussion of magnetic relaxation in lanthanide (Ln) based SMMs,^8,24^ which invariably possess electronic structures with the characteristic profile in Fig. 1;^7,9^ that is, interelectronic repulsion dominates so that the Hund's rule ground state is a well isolated Russell-Saunders term, which is then split by spin–orbit coupling to give a well-isolated manifold of total angular momentum *J*, which is then split by crystal field (CF, or ligand field) interactions into linear combinations of *m*_*J*_ states.^7,25^ The theories discussed herein are equally applicable to spin–phonon coupling in molecules with other metal ions, but the importance of the various mechanisms changes when the energy scales and electronic state multiplicities differ. Note also that while we use terms such as “spin–phonon”, “spin system”, “spin Hamiltonian”, *etc.*, “spin” should be interpreted as a general total angular momentum.

SMMs are zero-dimensional superparamagnets and there is no phase transition associated with their magnetic memory, unlike for bulk ferromagnets. Thus, their magnetic dynamics are determined solely by their magnetic relaxation rates, the timescale on which an ensemble returns to thermodynamic equilibrium;^3,4^ this process is often called spin-lattice relaxation and given the symbol *T*_1_, but herein we will use the symbol *τ*. These rates are determined experimentally by measuring the time dependence of the magnetisation, often as a function of an external parameter such as temperature or magnetic field.^9,26^ Although magnetic relaxation is an ensemble property, the underlying rates are determined by how the individual molecules exchange energy with their environment. Thus, the process of a sample returning to equilibrium is the result of a great number of possible spin–phonon interactions and relaxation pathways. There are three common mechanisms typically invoked in the interpretation of relaxation rate data (Fig. 1):^19^ (i) the Orbach process, in which relaxation occurs *via* a series of single spin–phonon absorption and emission events up and over the energy barrier; (ii) the Raman process, in which two phonons interact simultaneously with the SMM (*i.e.* a phonon is scattered in a collision with the molecule), where usually the transition between the ground doublet states is of most importance; and (iii) quantum tunnelling of the magnetisation (QTM) where relaxation occurs directly between the ground doublet states and does not involve absorption or emission of phonons (not shown in Fig. 1); QTM will not be discussed further herein.

This Tutorial Review will start with a discussion of the phonon degrees of freedom, followed by the basics of spin–phonon interactions, and then will discuss in detail how these ingredients can be combined to calculate magnetic relaxation rates under single-phonon (Orbach) and two-phonon (Raman) paradigms.

## 2 Phonons

The first point of discussion is the vibrational modes themselves, which are frequently referred to as the “bath” in the literature on open quantum systems, or the “lattice” in the context of physical chemistry. We begin with a discussion of the essential features of the vibrations of gas-phase molecules. An isolated molecule in the gas phase with *n*_a_ atoms has 3*n*_a_ degrees of freedom, made up of the three Cartesian directions *x*, *y* and *z* for each atom, which combine together to form translations, rotations and vibrations. The combinations of atomic displacements, and the associated vibrational frequencies (energies), are obtained within the harmonic approximation as the eigenvalues and eigenvectors of the mass-weighted Hessian matrix ***H***, which we refer to here as the “dynamical matrix” ***D***:1
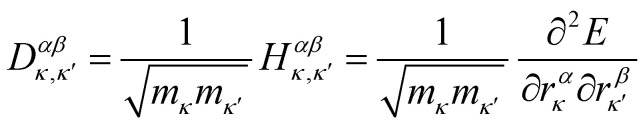
where *r*^*α*^_*κ*_ are the atomic degrees of freedom, the indices *κ*, *κ*′ and *α*, *β* label the atoms and Cartesian directions, respectively, and *m*_*κ*_ are the atomic masses.

**Fig. 2:**
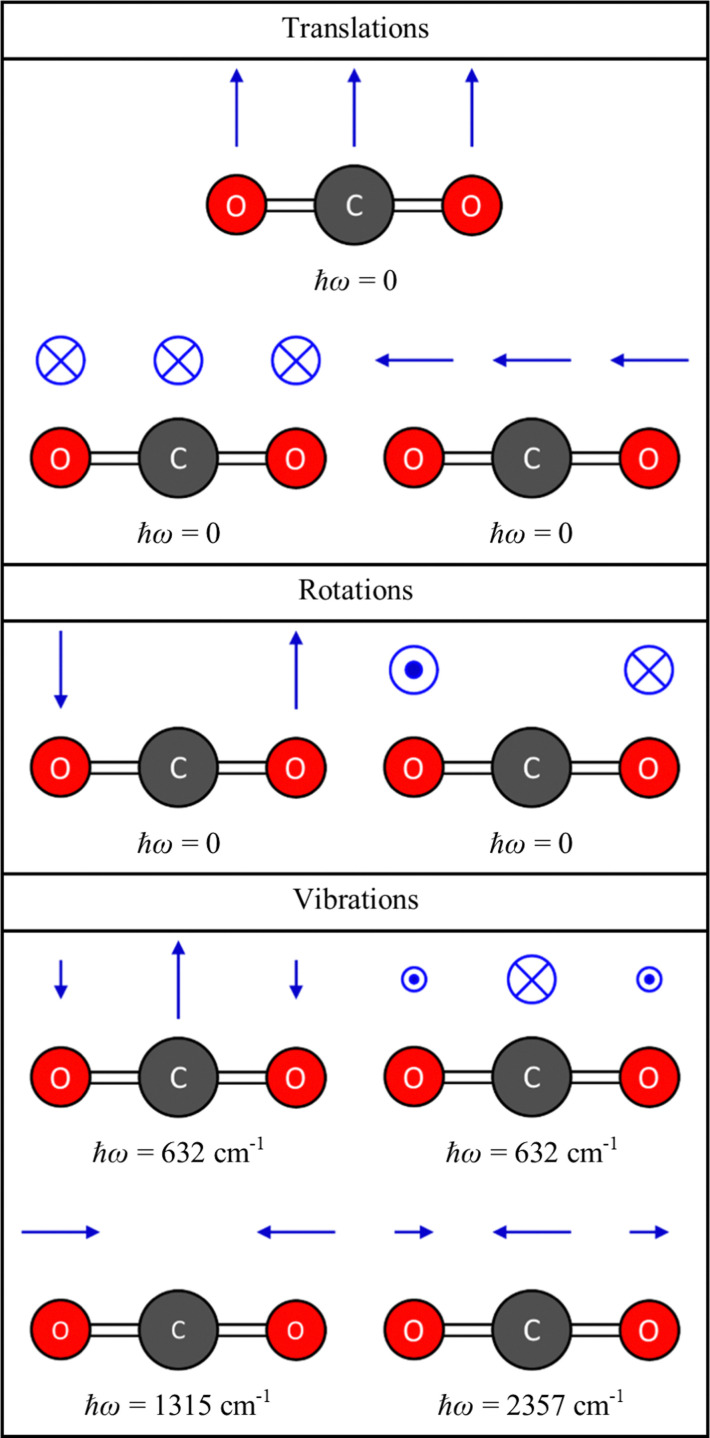
Energies and displacement vectors for the translations, rotations and vibrations of the CO_2_ molecule, obtained by diagonalising the dynamical matrix ***D*** defined in eqn (1), from DFT calculations (see ESI,† for details). The energies are obtained by taking the square root of the eigenvalues *ω*^2^ and multiplying by the reduced Planck constant *ħ*, and the displacements are obtained from the eigenvectors ***W*** using eqn (3) with a unit amplitude *Q* = 1.

***D*** is a 3*n*_a_ × 3*n*_a_ matrix, and diagonalisation yields 3*n*_a_ squared frequencies *ω*^2^ (eigenvalues) and associated mass-weighted displacements ***W*** in the form of 3*n*_a_-component vectors (eigenvectors). Fig. 2 shows the energies and displacements obtained by diagonalising the dynamical matrix of CO_2_, calculated with density–functional theory (DFT; see ESI,† for details). Of the 3*n*_a_ combinations of the degrees of freedom, three combinations are rigid translations, and two or three are rigid rotations depending on whether the molecule is linear or non-linear. These collective motions of the atoms do not perturb the intramolecular interactions, and therefore have *ω*^2^ = 0. The remaining 3*n*_a_ − 5 or 3*n*_a_ − 6 combinations are the vibrations, which are mutually-orthogonal relative motions of the atoms that conserve the centre of mass but have an energy change associated with them (*i.e. ω*^2^ > 0). For CO_2_, there are three translations and two rotations with *ħω* = 0, and four vibrational modes comprising a doubly-degenerate bend (*ħω* = 632 cm^−1^) and symmetric and antisymmetric stretches (*ħω* = 1315 and 2357 cm^−1^, respectively).

**Fig. 3:**
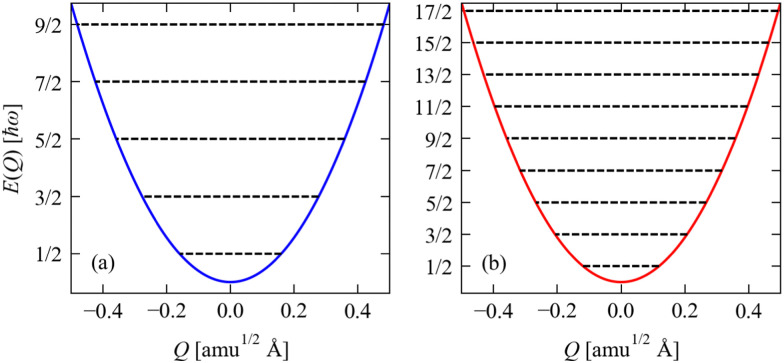
Potential energy as a function of amplitude *E*(*Q*) for the two stretch modes in CO_2_ determined from eqn (2): (a) the symmetric stretch with *ħω* = 1315 cm^−1^, and (b) the antisymmetric stretch with *ħω* = 2357 cm^−1^. The *E*(*Q*) for both modes are shown over a range of *Q* = ±0.5, and the energies are given in units of *ħω*. On each plot, the black lines show the harmonic energy levels *E*_v_ determined from eqn (4). Note that as the energies are given in units of *ħω*, the implication is that modes with small *ħω* will, in general, have larger displacements |*Q*| for a given vibrational quantum state than will modes with large *ħω*.

Within the harmonic approximation, the potential energy of the vibrational modes is quadratic in the displacement amplitude *Q* according to:2
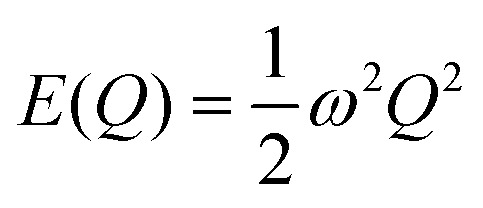
The relation between *Q* and the Cartesian displacement of the *κ*^th^ atom is given by:3
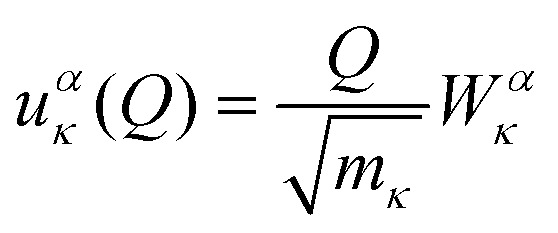
where *W*^*α*^_*κ*_ is the component of the eigenvector for the atom along the direction *α*. Solution of the Schrödinger equation for the kinetic energy and the potential energy function in eqn (2) yields allowed energy levels quantised into units of *ħω*:4
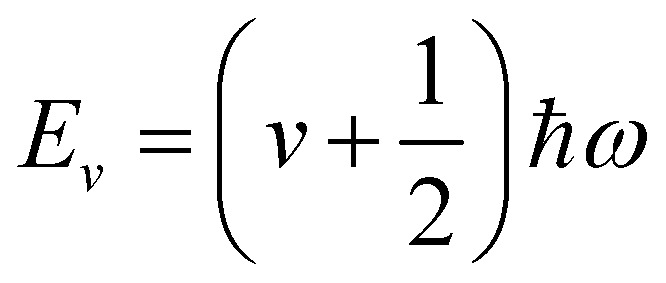
where *v* is the vibrational quantum number; *v* = 0 corresponds to the energetic ground state, and the residual 
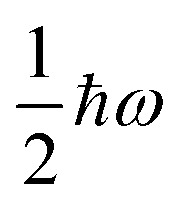
 of energy in this state is termed the “zero-point” energy of the vibrational mode. Fig. 3 shows the potential energy as a function of amplitude for the two stretching modes in CO_2_, together with the allowed harmonic energy levels.

We now consider the generalisation of the harmonic approximation to periodic crystals. Vibrations in crystalline solids, termed “phonons”, take the form of travelling waves of atomic displacements that propagate through a crystal. As in molecules, the relative atomic motion has an associated frequency *ω*, and the wavepacket propagates through the crystal with a defined wavelength *λ* and velocity *ν*.

In principle, the phonon modes could be obtained by constructing and diagonalising a dynamical matrix in the form of eqn (1) for the whole crystal. However, since *n*_a_ approaches Avogadro's number (*N*_A_ ≈ 6.022 × 10^23^), such a “brute force” approach is not feasible. Instead, the periodicity of the crystalline phase can be exploited to view a (macroscopic) crystal as an infinite repeating array of primitive unit cells with *n*_a_ atoms each. According to the Bloch theorem, a wavefunction *Ψ*(***r***) in such a periodic system can be expressed as the product of a cell-periodic function ***u***(***r***) and a plane-wave exp(*i****q***·***r***):^27^

**Fig. 4:**
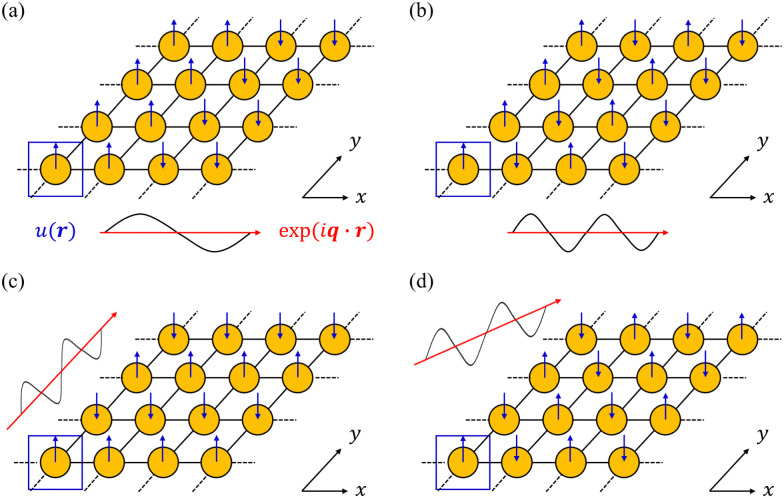
Schematic illustration of how the Bloch theorem defined in eqn (5) generates four of the phonon modes of a monoatomic 2D lattice. The displacement of the atom in the primitive cell defines the cell-periodic function *u*(***r***) (blue), which is replicated across the other unit cells in the lattice with a phase pattern defined by plane-wave terms exp(*i****q***·***r***) (red). In (a) and (b) the wavevector ***q*** is oriented along the real-space *x* direction with two different wavelengths corresponding to different modulation periods. In (c) and (d), the wavevector is aligned along the *y* and *xy* directions, respectively, with the same wavelength as in (b).

5 *Ψ*(***r***) = ***u***(***r***) × exp(*i****q***·***r***)

where ***q*** is a wavevector defined in the reciprocal space (first Brillouin zone, BZ) of the crystal. We note that wavevectors are conventionally discussed in “reduced” units of fractions of the reciprocal lattice vectors, which are related to the ***q*** used herein as ***q*** = 2π(*q*^R^_1_a* + *q*^R^_2_b* + *q*^R^_3_c*), where *q*^R^_*n*_ are the components of the reduced vector ***q***^R^, and a*, b* and c* are the reciprocal lattice vectors. In the context of a phonon, the ***u***(***r***) correspond to a set of relative atomic displacements in the primitive cell, and the wavevectors ***q*** in the plane-wave terms specify the propagation direction and wavelength of the phonons, and define how the local atomic displacements are modulated across the other unit cells in the crystal (Fig. 4).

Application of the Bloch theorem yields the modified dynamical matrix ***D***(***q***) given by:6
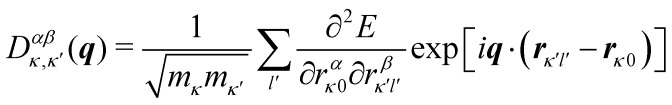
where the indices *l*, *l*′ label different crystallographic unit cells. Diagonalising a given ***D***(***q***) yields 3*n*_a_ squared frequencies *ω*_***q****j*_^2^ and corresponding mass-weighted displacements ***W***_***q****j*_ associated with the wavevector ***q***, where *j* is the phonon band index and runs from 1 to 3*n*_a_.

Use of the Bloch theorem simplifies the problem of determining the phonons in an infinite periodic crystal to diagonalising a set of ***D***(***q***) at a formally infinite number of wavevectors ***q***. However, the *ω*_***q****j*_ and ***W***_***q****j*_ typically do not vary significantly for small changes in ***q***, allowing physical properties requiring integrals over the BZ to be replaced by a finite, discrete sum. Furthermore, the phonon modes at certain wavevectors can be related by crystal symmetry operations, and most properties can therefore be obtained by considering only the subset of ***q*** that lie within the “irreducible” part of the BZ; for crystals in high-symmetry spacegroups, this is a fraction of the full BZ.

To discuss the phonon spectra of solids, two quantities are commonly used: the phonon dispersion (band structure) and the density of states (DoS). The dispersion *ħω*_*j*_(***q***) shows the evolution of the 3*n*_a_ phonon energies with the wavevector ***q*** along a specific path through the BZ, and is typically displayed with a path visiting the set of high-symmetry ***q***-vectors comprising the zone-centre ***q***^R^ = (0, 0, 0) (Γ) and the zone-boundary points, the number, coordinates and labels of which are defined by the crystallographic spacegroup. The DoS *g*(*ħω*) shows the relative number of modes as a function of phonon energy integrated over the BZ:7
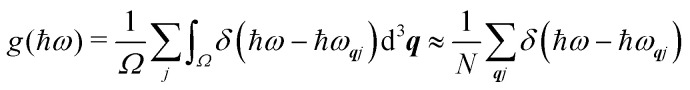
where *Ω* is the volume of the BZ, *δ* is the Dirac delta function (*δ*(*x*) = 0 for *x* ≠ 0 and 
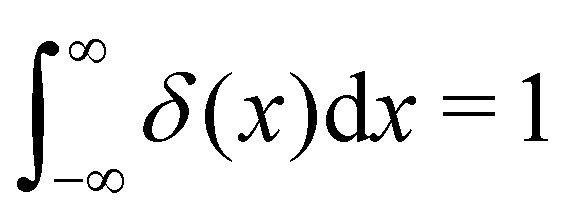
), and *N* is the number of wavevectors included in the summation approximating the integral. We note that the DoS is normalised so that it integrates to the number of modes in the primitive cell, *i.e.*
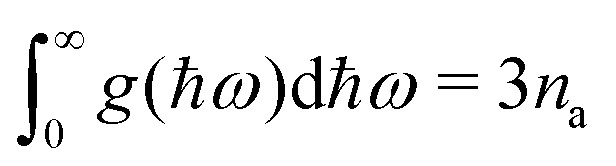
. It is also common to use the eigenvector coefficients to quantify the contributions from different atoms in order to construct an atom-projected partial DoS showing how atoms contribute to modes in different energy ranges.

**Fig. 5:**
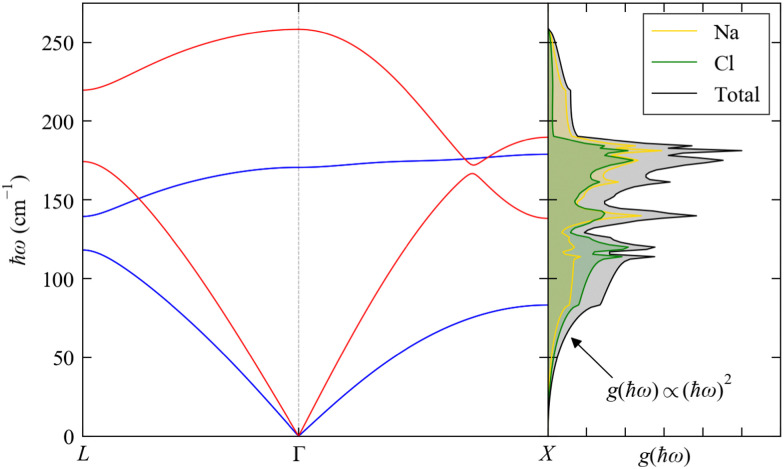
Phonon dispersion *ħω*_*j*_(***q***) and density of states *g*(*ħω*) (DoS) of NaCl from DFT calculations (see ESI,† for details). The dispersion is shown along the wavevector path 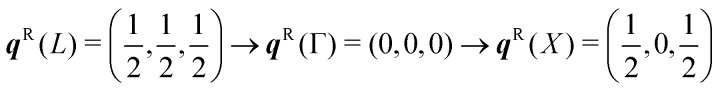. There are 3*n*_a_ = 6 bands at each ***q***, but the high symmetry of the cubic spacegroup means that a pair of acoustic modes and a pair of optic modes are degenerate along this path; the singly-degenerate bands are shown in red, and the doubly-degenerate bands in blue. The DoS is shown in black and the projections onto the Na and Cl atoms are shown in yellow and green, respectively.

The dispersion and DoS for NaCl (calculated using DFT, see ESI†) highlight several features common to phonon spectra (Fig. 5). There are three “acoustic modes” that correspond to concerted motion of all of the atoms in the primitive cell in the same direction with the same amplitude; at ***q*** = Γ, this motion is fully in-phase for all unit cells in the crystal, resulting in three rigid translations of the entire crystal with *ħω*_*j*_ = 0 (one of the acoustic modes for NaCl is doubly degenerate along the dispersion path). [We note that, unlike for gas-phase molecules, rigid rotations of crystals are not defined.] At the zone-boundary wavevectors, here 
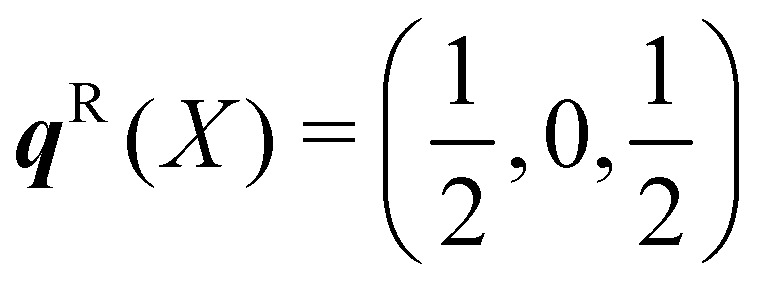
 and 
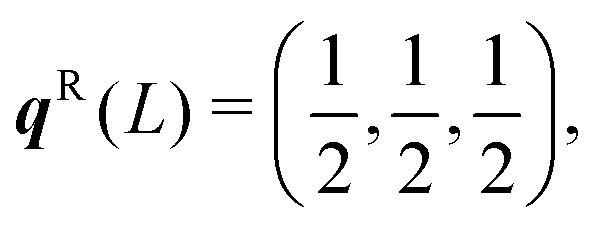
 the atomic motion in neighbouring unit cells is fully out-of-phase along particular real-space directions, and therefore the energy of the acoustic modes increases sharply away from Γ with an approximately linear dispersion. This linear dependence at small |***q***| leads to an approximately quadratic increase in the low-energy DoS,^27^ which is the basis for the Debye approximation common in traditional texts on spin–phonon coupling.^22^ The acoustic modes have frequencies in the Hz–kHz range at ***q*** ≃ Γ and allow the transport of sound waves through the crystal, which is the origin of their name. The slope of the dispersion ∂*ω*_***q****j*_/∂***q*** determines the “group velocity” ***v***_***q****j*_ of the modes, which gives the velocity and direction with which the modes travel through the crystal. For NaCl, the linear dispersion of the acoustic modes near Γ results in these modes having the largest |***v***_***q****j*_|, which sets the speed of sound propagation through the crystal.

The remaining 3*n*_a_ − 3 modes involve opposing motions of the atoms in the unit cell, and thus have *ħω*_*j*_ > 0 at all ***q***. These modes can, in principle, lead to a change in electric polarisation (the solid-state analogue of the dipole moment) and/or electric polarisability within the primitive cell, and thus may interact with light; they are therefore termed “optic modes”. Approximately two-thirds along the path between Γ and *X* in the phonon dispersion of NaCl (Fig. 5) there is an avoided crossing between an acoustic and optic branch, indicating mixing between these modes; at wavevectors away from Γ, the distinction between these two classes of modes is therefore not always clear. The DoS of NaCl shows a continuous spectrum of modes up to ∼250 cm^−1^, and the partial DoS shows that Na and Cl contribute roughly equally across the full range of modes. This is due to their similar mass (*m*_Na_ = 22.99 and *m*_Cl_ = 35.45 amu), and in systems with large mass differences between the constituent atoms the DoS often shows distinct energy regions where a particular type or group of atoms dominates the vibrations.

**Fig. 6:**
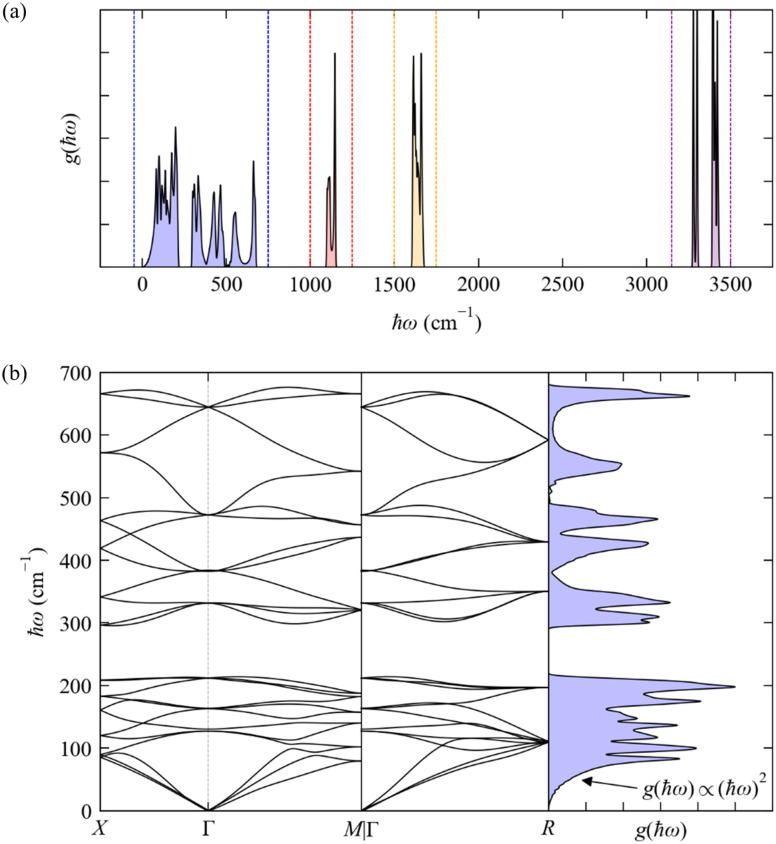
Phonon dispersion *ħωj*(***q***) and density of states *g*(*ħω*) (DoS) of cubic crystalline NH_3_ from DFT calculations (see ESI,† for details). Panel (a) shows the full DoS and highlights four distinct groups of modes, *viz.* the “external” (pseudo-)acoustic modes formed from rigid-molecule translations and rotations (blue), and the “internal” modes formed from the NH_3_ bending (red), H–N–H “scissoring” (orange), and N–H symmetric and antisymmetric stretch vibrations (purple). Panel (b) shows the phonon dispersion and DoS from 0–700 cm^−1^ corresponding to the blue region in (a).

The phonon spectra of molecular crystals present some additional noteworthy features, which we illustrate here with the phonon dispersion and DoS of the cubic phase of crystalline NH_3_ (Fig. 6; calculated using DFT, see ESI†). In the general case where there are multiple molecules in the primitive cell, the translations and rotations of each molecule (*i.e.* those that are present in the gas phase) combine to produce low-energy phonons involving various rigid-body motions of whole molecules that are often termed “external modes”. Three combinations of these translations preserve the intermolecular distances and give rise to the acoustic modes with *ħω*_*j*_ = 0 at ***q*** = Γ as in inorganic crystals. Other combinations of translations, rotations, and translations/rotations have non-zero energy, but are still relatively low-energy motions due to the generally weaker intermolecular interactions, and hence there are additionally a large number of low-energy dispersive modes that mix with the acoustic modes at ***q*** ≠ Γ; we refer to this loosely-defined set of modes as “pseudo-acoustic” modes.

As a non-linear molecule, NH_3_ has three translations, three rotations and six vibrations in the gas phase. There are four NH_3_ molecules in the primitive cell of the cubic crystal structure (*n*_a_ = 4 × 4 = 16) and hence 48 phonon modes at each wavevector ***q***. The rigid-molecule translations and rotations combine to form a dense group of 24 phonon bands spanning a range of 0–700 cm^−1^ (Fig. 6). In this case the translations and rotations are separated by a small gap of ∼100 cm^−1^ between the 12 translational (pseudo-)acoustic modes from around 0–200 cm^−1^, and the 12 rotational pseudo-acoustic modes from ∼300–700 cm^−1^. Both sets of phonons are pure external modes, and the wide dispersion is due to the H-bonding network formed between the molecules in the unit cell. The low-energy DoS remains approximately quadratic as in the Debye approximation, as the region where *ħω*_*j*_ → 0 is dominated by the acoustic modes, but the DoS ceases to behave quadratically above the “Debye limit” due to the pseudo-acoustic modes.^28^

The mid- and high-energy modes in molecular solids tend to be made up of combinations of intramolecular vibrations (“internal modes”). Particularly at higher energies these tend to be localised vibrations (*e.g.* bond stretches) that often do not significantly disrupt the intermolecular interactions, resulting in a weak dependence on ***q*** and hence flat (“dispersionless”) bands and sharper features in the DoS. For cubic NH_3_, there are three distinct groups of internal modes (Fig. 6a) corresponding to combinations of the bending modes (1100–1150 cm^−1^, four modes), scissoring modes (1600–1700 cm^−1^, eight modes), and the symmetric and antisymmetric stretches (3250–3450 cm^−1^, 12 modes). The two types of N–H stretches do not mix, and the higher-energy group of stretching modes is notably split into two distinct features centred around 3290 and 3400 cm^−1^, with four and eight modes, respectively. The features corresponding to the internal modes are much narrower than those from the external modes, indicating a narrower energy dispersion.

To conclude this section, we cover three more aspects of phonons that are relevant to the material in the following sections. Firstly, while the phonon energies are quantised into units of *ħω*_***q****j*_ as in the gas phase, for most purposes it is the average harmonic energy level of each mode that is of interest, which is determined by the Bose–Einstein occupation number *n̄*_***q****j*_:8
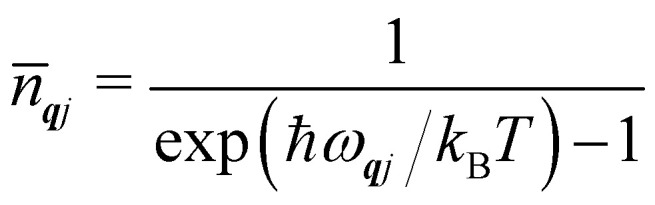


Secondly, the analogous expression for the atomic displacements as a function of the mode amplitude in eqn (3) for phonons in solids is:9
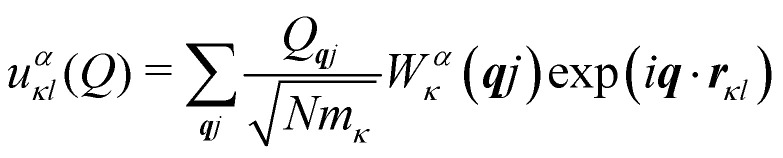
where the factor of 
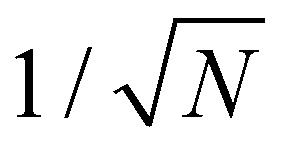
 is required to maintain the relation in eqn (2). This can be used to compute the coupling between phonon modes and other material properties, in particular the CF parameters (CFPs), as discussed in the following sections.

Finally, in the harmonic approximation the phonon modes are strictly independent oscillators and therefore have infinite lifetimes *τ*_***q****j*_. However in real crystals, the phonon modes interact with one-another *via* collisions and decay, which give rise to finite lifetimes. As a consequence of the uncertainty principle, the phonons therefore have finite linewidths *Γ*_***q****j*_ = *ħ*/*τ*_***q****j*_ (full-width-at-half-maximum in energy units). [*n.b.* The symbol used for the phonon linewidths *Γ*_***q****j*_ should not be confused with the centre of the BZ ***q*** = Γ.] In the case of lifetime broadening such as this, the phonon lineshape is Lorentzian (eqn (10)). However, as the phonon DoS (eqn (7)) must go to zero at zero energy (*i.e. g*(0) = 0), the anti-Lorentzian lineshape should be used instead (eqn (11)). In practice, however, there is little difference if Lorentzian or even Gaussian lineshapes are used.10
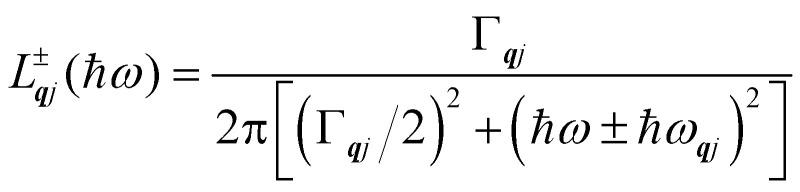
11
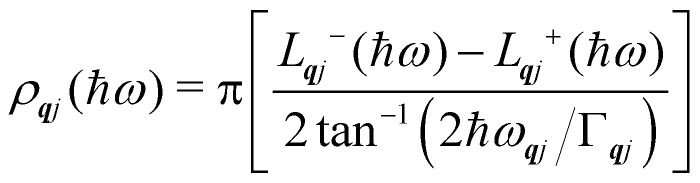


Phonon–phonon scattering is typically the main contributor to the lifetimes *τ*_***q****j*_ in semiconductors and insulators, although in some systems coupling between the electrons and phonons can also be important.^29^ The lowest-order scattering processes that conserve energy and (crystal) momentum are three-phonon processes; these can be calculated and used in conjunction with the harmonic phonon frequencies and eigenvectors to compute the *τ*_***q****j*_ from first principles,^30^ albeit at a considerable computational cost, especially for large systems such as molecular crystals. Hence, phonon linewidths are often treated as parameters^31^ or by using statistical approximations.^32^ However, we have recently shown that there is little impact on the spin dynamics when either fixed, approximated, or calculated linewidths are used, provided the BZ is integrated with a sufficiently dense ***q***-point grid.^33^

## 4 Electronic structure of lanthanide complexes

Now we turn to the spin states of the molecule, where herein we focus on the low-lying electronic states of Ln complexes. For most cases, the trivalent Ln^3+^ oxidation state is most relevant, with a well-isolated ground configuration 4f^*n*^5s^2^5p^6^; the 6s and 5d may become relevant depending on the oxidation state and the ligands surrounding the metal.^34^ Trivalent Ln^3+^ ions are unique in the periodic table in that their valence electrons reside in the 4f orbitals which are strongly contracted and shielded beneath a filled set of 5s and 5p orbitals. Given the inert nature of the 4f orbitals, the electronic structures of Ln^3+^ ions in molecules are well-described by the free-ion Hunds rule ground term ^2*S*+1^*L*_*J*_, where *S* is the total spin angular momentum, *L* is the total orbital angular momentum, and *J* = *L* ± *S* is the total angular momentum after spin–orbit coupling (the ± sign refers to either >7 or <7 electrons in the 4f shell). While there are exceptions (*e.g.* Sm^3+^ and Eu^3+^), for most Ln^3+^ ions the population of excited *J* multiplets is essentially zero at room temperature and below, which is the temperature range of interest for SMMs. Coordination of a set of ligands to a Ln^3+^ ion gives rise to a CF splitting of the 2*J* + 1 *m*_*J*_ states of the ground *J* multiplet, and is described by the CF Hamiltonian (eqn (12)).^35^12
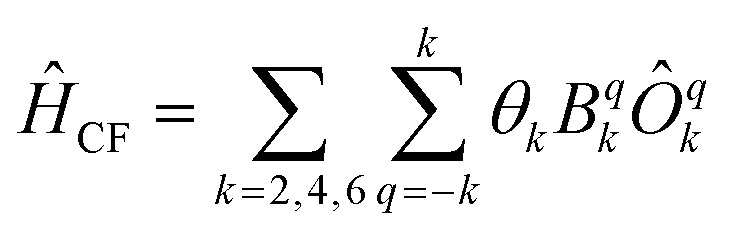


Here, *B*^*q*^_*k*_ are the Stevens CF parameters of rank *k* and order *q*, the *Ô*^*q*^_*k*_ are Stevens operator equivalents in the |*J*,*m*_*J*_〉 basis which are polynomials of the angular momentum operators *Ĵ*_*x*_, *Ĵ*_*y*_, *Ĵ*_*z*_, and the *θ*_*k*_ are operator equivalent factors which relate the multi-electron CF Hamiltonian in the |*J*,*m*_*J*_〉 basis to the single-electron CF Hamiltonian in the Slater determinant basis.^36,37^

## 5 Spin–phonon coupling

Under an initial approximation that the electronic spin system is non-interacting with the phonons, we can define the uncoupled equilibrium Hamiltonian (eqn (13)). Here, the electronic part is the CF Hamiltonian and the phonon part is the harmonic oscillator Hamiltonian; note the distinction between the phonon wavevector ***q*** and the order of the CF operator *q*.13
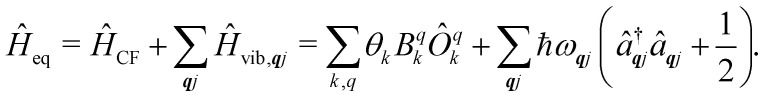


The operators *â*_***q****j*_ and *â*^†^_***q****j*_ denote phonon annihilation and creation operators, which act as 
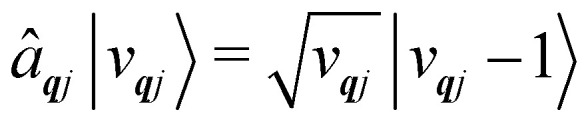
 and 
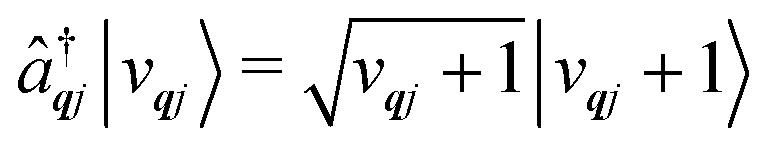
 and represent reducing or increasing the vibrational quantum number of phonon mode ***q****j*. These allow us to define the dimensionless mass- and frequency-weighted normal coordinates as^27^14
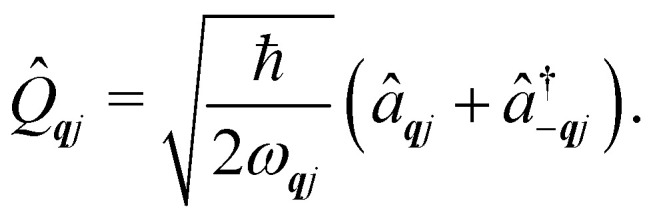
We note that the *Q̂*_***q****j*_ operator in eqn (14) is non-Hermitian, unless ***q*** coincides with the Γ point or at special points on the boundary of the BZ (see ESI†). This occurs because normal mode displacements are in general complex quantities characterised by amplitude and phase. The Hamiltonian in eqn (13) can be written in the direct (or tensor) product basis 
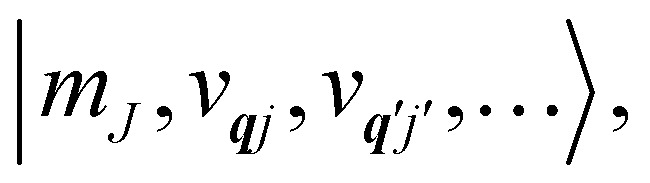
 and is solved by diagonalisation to give eigenstates 
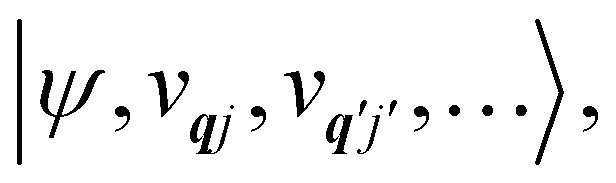
 where *ψ* denotes an electronic eigenstate and 
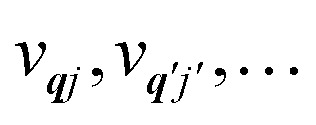
 are non-negative integers specifying the occupation number of each phonon mode.

The fundamental nature of the spin–phonon interaction considers how vibrations modulate electronic states. To this end, we describe spin–phonon coupling for the case of Ln SMMs using a modified form of the CF Hamiltonian, where the parameters *B*^*q*^_*k*_ are dependent on the displacement along the vibrational mode coordinates *Q*_***q****j*_ (eqn (15)). In the case of a transition metal, one may alternatively define the coupling Hamiltonian to arise from vibrational modulation of hyperfine coupling, the *g*-matrix, or zero-field splitting, for example;^32,38^ however for 
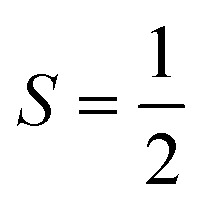
 systems, it is important to account for changes in the nature of the basis states that is not accommodated in a simple *g*-matrix model.^39^ Though in any case, we note that a model Hamiltonian need not be assumed and that the derivatives of the Hamiltonian matrix elements can be determined directly and employed in a similar vein.^40,41^ In eqn (15) we describe the modulation of the CF parameters using a Taylor series centred at the equilibrium structure *Q*_***q****j*_ = 0, where *B*^*q*^_*k*_(0) are the same equilibrium CF parameters appearing in eqn (13) and thus are not relevant for spin–phonon coupling. Thus, we can define a spin–phonon coupling Hamiltonian *Ĥ*_SP_ up to second-order as eqn (16), where *V̂*^(1)^_*qj*_ and 
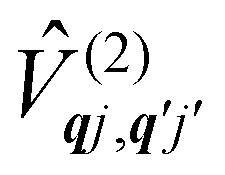
 describe first- and second-order spin–phonon coupling, respectively.15
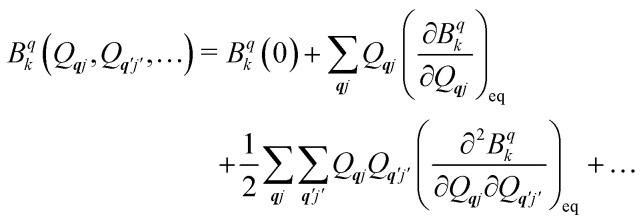
16
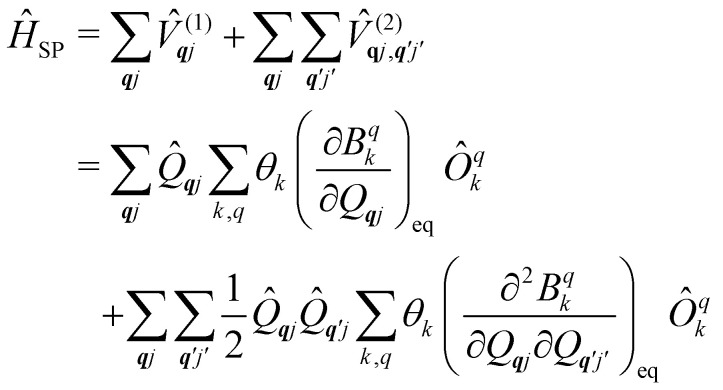
We note that, as a Hamiltonian operator, *Ĥ*_SP_ in eqn (16) must be Hermitian. However, due to the non-Hermitian definition of *Q̂*_***q****j*_ in eqn (14), this implies that the derivatives in eqn (16) are taken using complex-valued displacements along the normal mode coordinates.^14^ Alternatively, one can choose to re-write eqn (16) such that each term in the sum over ***q****j* are all individually Hermitian; we do this by introducing a new set of creation/annihilation operators *b̂*_***q****j*_ as linear combinations of *â*_***q****j*_ and *â*_−***q****j*_, and defining Hermitian dimensionless normal displacement operators as *X̂*_***q****j*_ = *b̂*_***q****j*_ + *b̂*^†^_***q****j*_ (see ESI†). For the rest of this review, we only consider the Hermitian representation of the normal displacements in terms of *b̂*_***q****j*_ and *b̂*^†^_***q****j*_, denoting their corresponding number states as |*v*_***q****j*_〉.

Eqn (16) suggests that the magnitude of the spin–phonon coupling is dependent on the number of ***q*** points included in the sum within the BZ, as the sums over ***q*** collect a growing number of terms. This apparent paradox is resolved by considering eqn (9) which includes a factor 
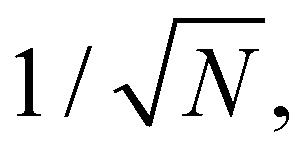
 thus decreasing the nuclear displacement amplitudes, and hence the coupling of individual modes, when the sum over ***q*** increases, resulting in size-consistent and convergent behaviour under these definitions.

The spin–phonon coupling Hamiltonian is evaluated in the product 
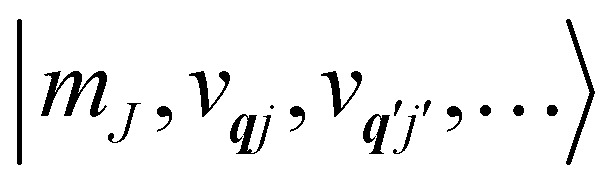
 basis, and for the first order term *V̂*^(1)^_***q****j*_ the non-zero matrix elements are of the form:17
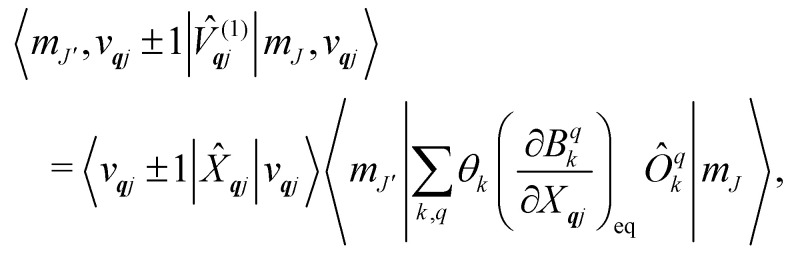
where mode ***q****j* has either gained or lost a quantum of vibrational energy. Evaluation of the electronic part is straightforward as it is simply a matrix element of CF operators. The vibrational matrix element can be evaluated with reference to the definition of the position operator in the basis of harmonic eigenstates, giving:18
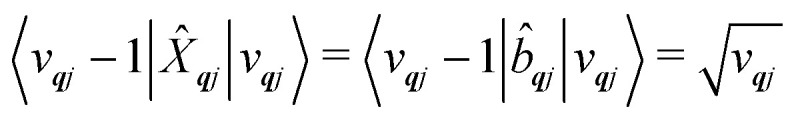
19
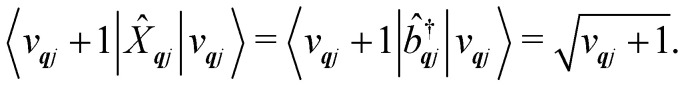
Considering the second-order coupling 
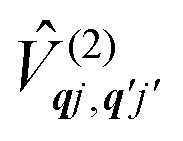
 with modes ***q****j* and ***q***′*j*′, the non-zero matrix elements are given by:20
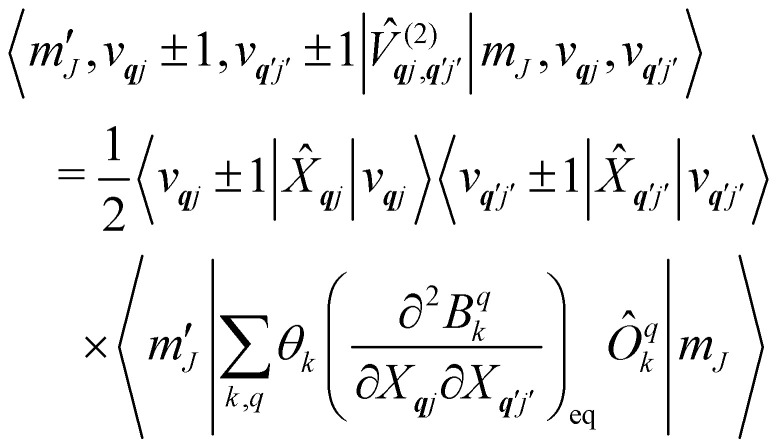
and21
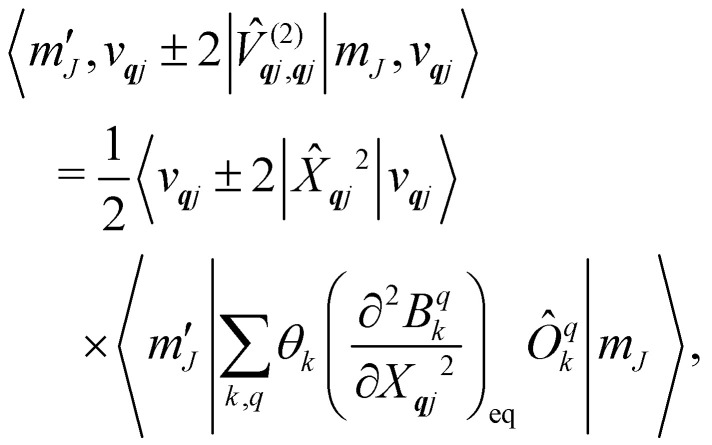
where22
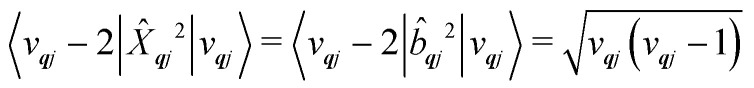
23
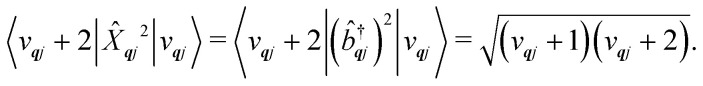
We will see in Section 6 that crucial quantities for determining the spin dynamics turn out to be the square modulus of the matrix elements of *Ĥ*_SP_ (eqn (17) and (20)), which consist of an electronic and a vibrational component. When considering the vibrational terms |〈*v*_***q****j*_ ± 1|*X̂*_***q****j*_|*v*_***q****j*_〉|^2^, we will be concerned with cases where the phonon modes are in thermal equilibrium; *i.e.* the *v*_***q****j*_ harmonic vibrational levels are statistically occupied with a probability proportional to the Boltzmann factor 
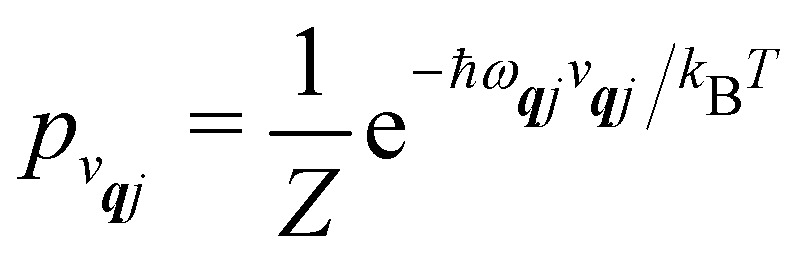
 (where *Z* is the partition function 
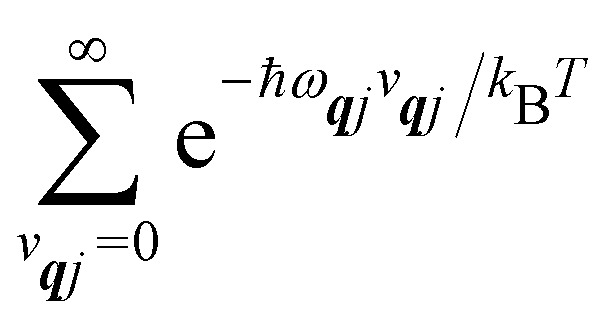
). Thus, averaging over thermally populated vibrational levels, gives24
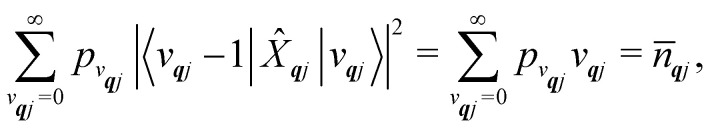
25
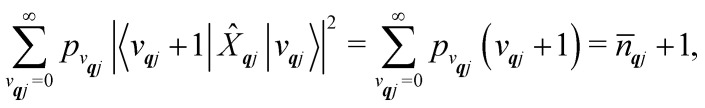
where *n̄*_***q****j*_ is the Bose–Einstein occupation number defined in eqn (8). While calculating the vibrational and electronic component of the matrix elements of *Ĥ*_SP_ is straightforward, obtaining the spin–phonon coupling parameters 
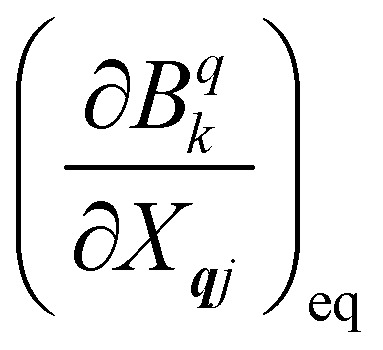
 and 
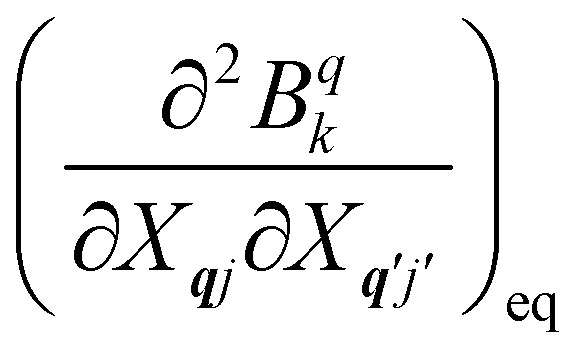
 is not straightforward; see Section 7. With the total Hamiltonian of a coupled spin and phonon system up to second-order in the spin–phonon coupling defined as *Ĥ*_T_ = *Ĥ*_eq_ + *Ĥ*_SP_, one may examine how the spin–phonon coupling affects molecular properties. For instance, we could calculate vibronic spectra directly in the coupled spin-vibrational basis,^13,42^ or we could calculate how the phonon bath leads to magnetic relaxation of the electronic spin system;^14,31^ the latter will be our focus here.

## 6 Spin dynamics and magnetic relaxation

In order to discuss magnetic relaxation, we must consider how the spin and phonon systems evolve in time and influence one-another; from here we will refer to phonons as “the bath” interchangeably. The goal of our interrogation is to assess the probability that a spin–phonon interaction leads to a spin flip in the electronic system, *i.e.* magnetic relaxation. In general, the dynamics of the phonon bath is assumed to be much faster than the spin dynamics of the SMM (though this assumption does not have to be made, see below), and the overall phonon–molecule interactions leading to a spin flip include: (i) phonons are absorbed by the SMM, (ii) phonons are emitted by the SMM, and (iii) phonons are scattered inelastically by the SMM. This section is separated into two subsections: in the first, we discuss open quantum systems methods which contain the most accurate approaches for dealing with the dynamics of quantum systems, starting from a minimal set of approximations and relaxing the accuracy of our approach. In the second, we discuss methods based on perturbation theory, which are the traditional approaches taken in this field, relying on a set of approximations from the outset.

### 6.1 Open quantum systems methods

As we are concerned with calculating the spin-flip probabilities, we must work with probabilistic measures of the quantum states; this necessitates use of the density matrix, *ρ̂*. The density matrix encodes the probabilities of finding the total system in a particular state (diagonal matrix elements) and also allows us to describe quantum coherences between states (off-diagonal matrix elements); note that here the states of the total system include both spin and phonon degrees of freedom. At the most exact level of theory, we could construct a density matrix representation of the spin-vibrational system described by the total Hamiltonian *Ĥ*_T_ starting in a well-defined state, *e.g. ρ̂*(0) = |*Ψ*_spin–vib_〉 〈*Ψ*_spin–vib_|, and consider its unitary evolution according to the Liouville–von Neumann equation *ρ̂*(*t*) = e^−*iĤ*_T_*t*/*ħ*^*ρ̂*(0)e^*iĤ*_T_*t*/*ħ*^ (the analogue of the time-dependent Schrödinger equation).^43^ Performing such a calculation would include all interactions between the spin and phonons and give us the knowledge of the system at all times. However, this is prohibitively expensive in practice owing to the very large number of phonon modes in any real system, and hence there have arisen many ways to find approximate solutions to this problem: this is a field of research called open quantum systems.^44–47^ The first simplification in solving this problem is to identify that we are not very interested in the state of the bath in the end, rather, we only care about the probability of the SMM undergoing a spin flip. Hence, we can perform a partial trace of *ρ̂* over the phonon modes: this is a mathematical operation to remove the phonon degrees of freedom from the total density matrix by averaging over them (often referred to as “tracing out the bath”) to give the reduced density matrix of the spin system:26
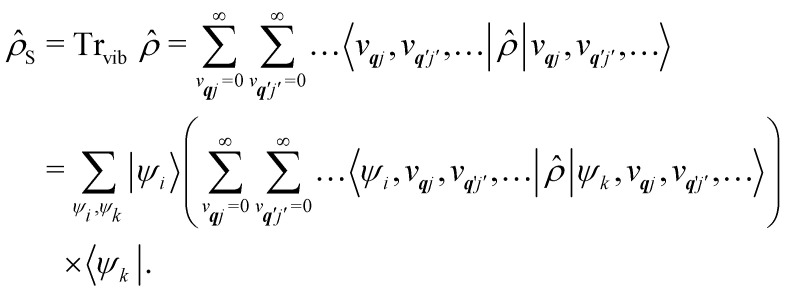
Note that the expectation values of *ρ̂* in the above equation are taken with respect to the phonon degrees of freedom only, leaving us with a reduced density matrix for the spin system. This is made evident in the second line of eqn (26), where the matrix elements of *ρ̂*_S_ in the electronic eigenbasis are given explicitly in terms of the matrix element of *ρ̂*. This procedure could be done either before or after interrogation of spin system dynamics. For instance, chain-mapping methods such as time-evolving density using orthogonal polynomial algorithm (TEDOPA)^48,49^ employ a linearisation of the bath Hamiltonian to approximate the dynamics of the whole system with density matrix renormalisation group (DMRG) methods,^50^ after which a trace over the bath degrees of freedom gives the desired reduced density matrix. Alternatively, another class of methods aims at finding approximations for the time evolution of the reduced density matrix of the spin system alone (eqn (26)) after performing the partial trace over the bath degrees of freedom analytically. In this class we find methods such as the quasi-adiabatic path integral (QUAPI),^51,52^ hierarchy equations of motion (HEOM),^53,54^ or the time-evolving matrix product operator (TEMPO).^55^ While all of the above are examples of methods that can approach high-degrees of accuracy that may be required when the spin and phonon systems are strongly coupled, in the domain of molecular magnetism it is usually safe to assume that the spin and phonon systems are weakly coupled. Moreover, magnetic relaxation is typically much slower than the underlying phonon dynamics, thus allowing for a clear separation of system and bath timescales; we have recently confirmed this separation of timescales by calculating the phonon lifetimes for a molecular crystal of a Dy(iii) SMM.^33^ These additional constraints simplify the problem considerably, allowing one to treat the phonons as a weakly coupled bath with no memory (on the timescale of the spin system). Under these assumptions, commonly referred to as the Born–Markov approximation, the dynamics of the reduced density matrix is described by the Redfield equation:^44,56^27
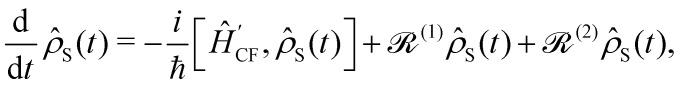
where the square brackets denote the commutator ([*Â*,*B̂*] = *ÂB̂*−*B̂Â*), and 
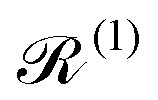
 and 
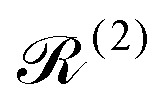
 are the Redfield superoperators arising from the first- and second-order spin–phonon coupling in eqn (16). Denoting the electronic part of the first- and second-order spin–phonon coupling operators as:28
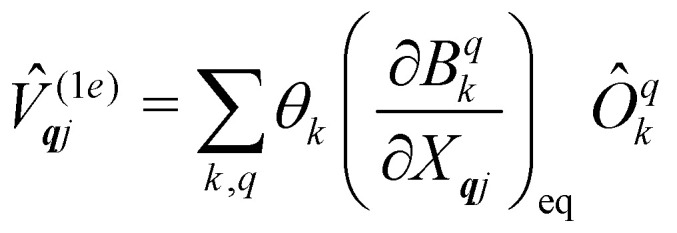
29
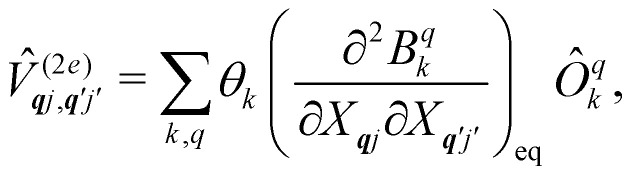
the Redfield superoperators can be written as:30
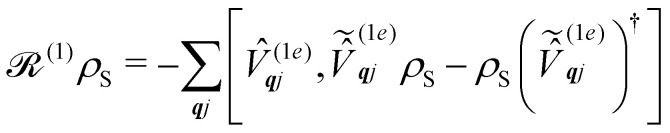
31
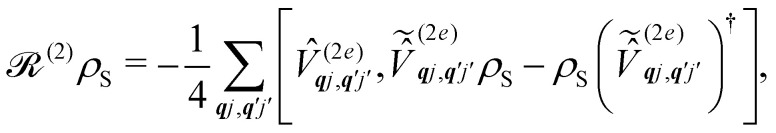
where:32
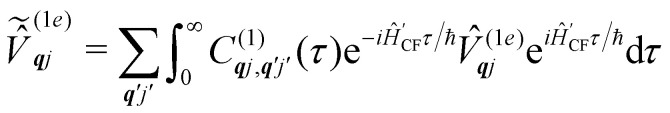
33
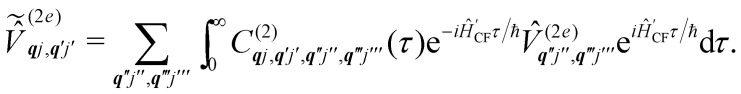
The time-dependent coefficients in eqn (32) and (33) are defined as34
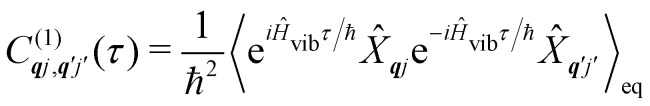
35
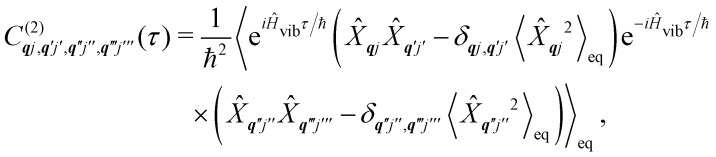
where 
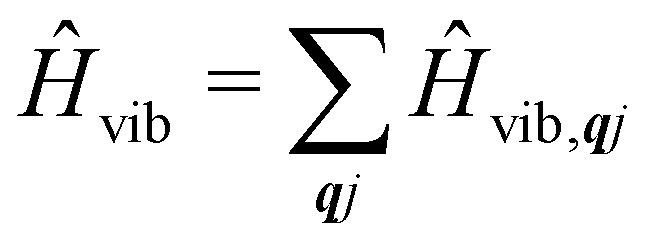
 is the total vibrational Hamiltonian in eqn (13). Eqn (34) and (35) represent the bath correlation functions calculated for a thermal distribution of phonons (eqn (8)). The thermal averages over phonons appearing in eqn (34) and (35) can be calculated as 〈…〉_eq_ = Tr_vib_{…*ρ̂*_eq_}, where *ρ̂*_eq_ = e^−*Ĥ*_vib_/*k*_B_*T*^/Tr_vib_{e^−*Ĥ*_vib_/*k*_B_*T*^} is the thermal density matrix for phonons. The CF Hamiltonian appearing in eqn (27) includes a correction arising from the second-order spin–phonon coupling, 
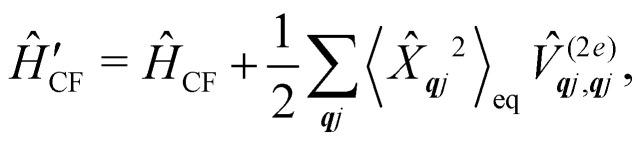
 accounting for the average energy shift induced by the quadratic terms.

The Redfield superoperators 
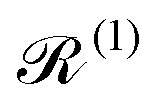
 and 
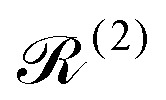
 in eqn (27) describe one- and two-phonon processes corresponding to the linear and quadratic terms of the expansion in eqn (16) (*i.e.* Orbach and Raman-II processes; see Section 6.2). In fact, Redfield theory incorporates the effect of the spin–phonon coupling in eqn (16) only to lowest order; higher-order interactions, *i.e.* two-phonon processes induced by the linear spin–phonon coupling (Raman-I mechanism) require either extending Redfield theory or resorting to other strategies that neglect the influence of electronic coherences (see Section 6.2 and ESI†).^14^ In principle, the effect of arbitrarily high-order spin–phonon interactions on the dynamics of the reduced spin density matrix *ρ̂*_S_ can be included systematically in a quantum master equation of a similar form to eqn (27)*via* the time-convolutionless projection operator method,^44^ which amounts to expanding the reduced system dynamics in powers of the system-bath coupling, giving further corrections to the equation of motion for *ρ̂*_S_ (eqn (27)). In practice, however, going beyond second-order is not very common for systems with more than a few electronic states or with highly structured vibrational environments, since the calculation of higher-order contributions to the dynamics will likely require computational resources comparable to the ones required for numerically exact methods (such as the ones mentioned above), which are more generally applicable, since they do not rely on a perturbative expansion in the system-bath coupling. However, the Redfield equation (and its secular version) remain extremely valuable tools to investigate the dissipative dynamics of quantum systems, especially when used in conjunction with other prescriptions to extend its validity beyond the Born–Markov approximation. A common strategy of this type is to redefine the boundary between system and bath by partitioning the total Hamiltonian *Ĥ*_T_ = *Ĥ*_eq_ + *Ĥ*_SP_ in a different way, in general such as 
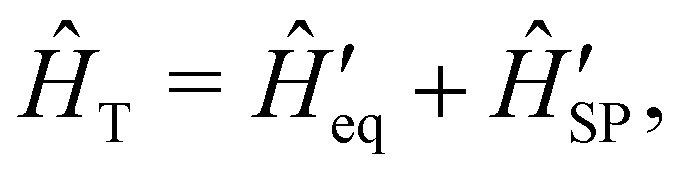
 where the revised system Hamiltonian 
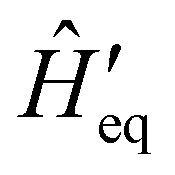
 now contains the “important” spin-bath dynamics and the residual system-bath coupling 
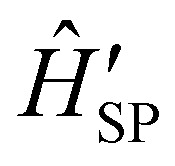
 is ammenable to description with the Born–Markov approximation. As an example, the reaction coordinate mapping method^57^ aims to capture non-Markovian effects on the electronic system dynamics by identifying a single collective coordinate of the bath, given by a suitable linear combination of individual coordinates *X̂*_***q****j*_, which is treated exactly, while the rest of the bath, which only couples to the collective mode, is treated with Redfield theory. Different criteria for selecting the most relevant bath pseudo-modes may be chosen, leading to different methods.^58,59^ Another strategy is to describe the coupled spin–phonon system in terms of polarons, displacing the phonon coordinates conditionally on the state of the spin system.^45^ The residual spin–phonon coupling in the polaron frame describes small harmonic displacements around the redefined equilibrium configurations, which can then be treated within Redfield formalism.^60,61^

We have discussed how the Redfield equation (eqn (27)) describes the dynamics of the reduced spin density matrix under the Born–Markov approximation, capturing the coupled time evolution of both electronic populations 〈*ψ*_i_|*ρ̂*_S_|*ψ*_i_〉 and coherences 〈*ψ*_i_|*ρ̂*_S_|*ψ*_k_〉 (where i ≠ k). A further approximation is often performed at this point, which amounts to averaging the oscillations induced by the time evolution of the electronic spin–phonon coupling operators in eqn (32) and (33). This procedure, known as the secular approximation,^44^ decouples the evolution of populations from coherences, and is justified when magnetic relaxation is much slower than the typical timescale of the unitary spin system dynamics, determined by energy differences between different eigenvalues of the CF Hamiltonian. Since SMMs exhibit either exactly or nearly doubly-degenerate electronic states (Fig. 1), this approximation is not always applicable, as the interaction between populations and coherences within a degenerate doublet cannot be decoupled, and other strategies must be used instead.^14^ If we decide to neglect electronic coherences entirely (which would not be appropriate if we cared about the decoherence of the quantum spin system induced by the phonons (*i.e.* the *T*_2_ timescale),^62^ but is usually a good approximation if we are only concerned with magnetic relaxation (*i.e.* the *T*_1_ timescale)), we can calculate the rates for a transition between two eigenstates of the CF Hamiltonian, *ψ*_i_ → *ψ*_f_ (given symbol *γ*_fi_), by taking the expectation value of the Redfield superoperators (eqn (30) and (31)) on the final state *ψ*_f_, and singling out the contributions from the terms 〈*ψ*_i_|*ρ̂*_S_|*ψ*_i_〉. For instance, applying this procedure to 
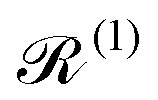
 gives:36
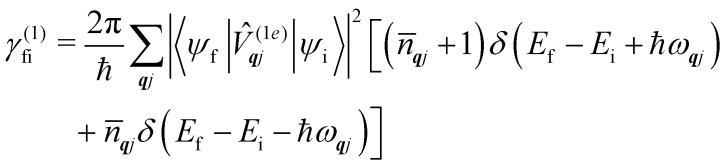
which describes single-phonon emission (arising from the first term in the square braces) or absorption (second term in the square braces) by the spin system. In the above, the phonon occupation numbers arise from expanding the mode coordinates *X̂*_***q****j*_ in terms of the phonon creation and annihilation operators (as performed in eqn (14) and considering the thermal averages (eqn (24) and (25)) where *n̄*_***q****j*_ is defined in eqn (8):

37 〈*b̂*_***q****j*_*b̂*^†^_***q****j*_〉_eq_ = *n̄*_***q****j*_ + 1

38 〈*b̂*^†^_***q****j*_*b̂*_***q****j*_〉_eq_ = *n̄*_***q****j*_.

In eqn (36), the phonon modes are considered in the harmonic limit as having infinitely-long lifetimes and hence the lineshape is given by the Dirac delta function; we could augment this expression by considering a finite linewidth, for example described by eqn (11).

Eqn (36) should be familiar to readers who have read traditional texts on magnetic relaxation or spin–phonon coupling: this is almost identical to the expression for single-phonon absorption or emission obtained by applying first-order time-dependent perturbation theory, *viz.* Fermi's Golden Rule, to the problem of magnetic relaxation; this is the route we take in the next section.

### 6.2 Perturbation theory methods

When we want to calculate the rates of magnetic relaxation in SMMs, we are not concerned with electronic coherences; if, on the other hand one wants to calculate decoherence rates, read Section 6.1. In this case, we can adopt a simpler theoretical framework and describe transitions between different states of the spin system using a classical rate equation. Furthermore, unlike for the single-phonon rate equation obtained from the first-order Redfield superoperator (eqn (36)), it is tedious to obtain rate-like equations for electronic populations considering the higher-order superoperators from the time-convolutionless expansion, and using perturbation theory (*viz.* Fermi's golden rule) is far more straightforward for this task.^14^ These two approaches lead to the same rate expressions, provided that some reasonable assumptions on the spin–phonon coupling strength and on the timescales of spin and phonon dynamics are made (see ESI† for a detailed derivation of one- and two-phonon rates). Using first-order time-dependent perturbation theory, the transition rate *γ*_fi_ between any two pairs of states |*ψ*_i_〉 and |*ψ*_f_〉 caused by a periodic perturbation *V̂* can be calculated as:^43^39
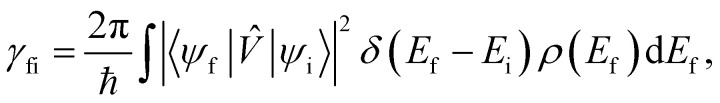
where *ρ*(*E*_f_) is the density of final states such that *ρ*(*E*_f_)d*E*_f_ is the number of final states in the energy range *E*_f_ → *E*_f_ + d*E*_f_. We first consider cases where the absorption or emission of a single phonon by the spin system drives transitions between electronic eigenstates |*ψ*_i_〉 and |*ψ*_f_〉 of the equilibrium electronic Hamiltonian (eqn (12)). For the case of magnetic relaxation, we only care about the states of the electronic spin system and not the bath states, and so we can perform the analogous procedure to “tracing out the bath” by first separating 〈*ψ*_f_|*V̂*|*ψ*_i_〉 into an electronic and vibrational part (*viz.*eqn (17) and (20)), then summing the contribution of all modes ***q**j* independently and assuming a thermal state of their phonon occupations (eqn (8)), which gives eqn (40) and (41), where the coupling operators are defined in eqn (28), *E*_i_ and *E*_f_ are the equilibrium electronic eigenstate energies. The only difference between this expression and eqn (36) is that we allow the phonon modes to have a finite linewidth, described by the lineshape *ρ*_***q****j*_ (eqn (11)); note that the integration variable *ħω* is an argument of the phonon occupation terms, the delta function, and the lineshape), and have explicitly separated the phonon absorption (*γ*_fi_^−^) and emission (*γ*_fi_^+^) terms.40
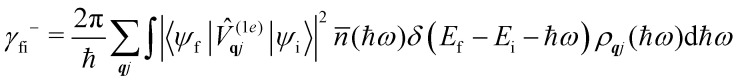
41
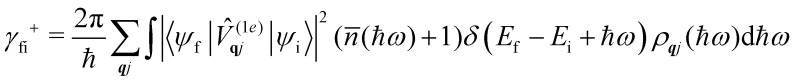
Since phonons have a finite linewidth, we allow their occupation numbers to vary continuously with the energy, *i.e. n̄*(*ħω*) = 1/[exp(*ħω*/*k*_B_*T*) − 1]. Note that for these single-phonon transitions, the integrals can be performed analytically to give only non-zero contributions where *ħω* = ±(*E*_f_ − *E*_i_), and we remind the reader that normalisation for the number of ***q***-points included in the summation is accounted for in the definition of the spin–phonon coupling coefficients in *V̂*^(1*e*)^_***q****j*_*via* the displacements in eqn (9). The approximation here is that the electronic spin–phonon coupling matrix element 〈*ψ*_f_|*V̂*^(1*e*)^_***q****j*_|*ψ*_i_〉 does not change over the phonon lineshape: this is a fair approximation for the case of narrow lines, but *n.b.* the zero-point displacement of a phonon with a different energy will be different from the central value, and this is not accounted for in this approximation.

Considering now two-phonon interactions, there are three possibilities: (i) two phonons are absorbed by the spin system (*γ*_fi_^−−^); (ii) two phonons are emitted by the spin system (*γ*_fi_^++^); or (iii) a phonon is scattered inelastically by the spin system (*γ*_fi_^−+^ and *γ*_fi_^+−^). Using first-order time-dependant perturbation theory as before, a similar derivation to the first-order rates can be carried out using the second-order spin–phonon coupling perturbation 
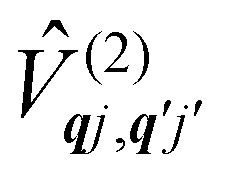
: this gives transition rates corresponding to the Raman-II mechanism (eqn (42)–(45)).^14,21^ Alternatively, one can use the first-order spin–phonon coupling Hamiltonian and take the time-dependent perturbation theory to second-order, thus defining the Raman-I mechanism (eqn (46)–(49)).^14,20,23^42
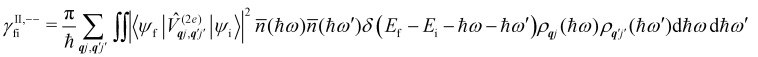
43
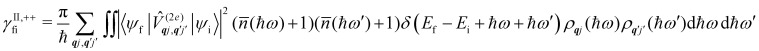
44
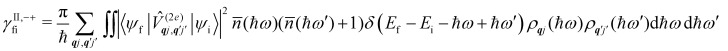
45
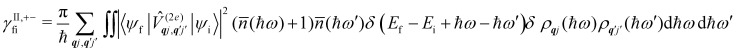
46
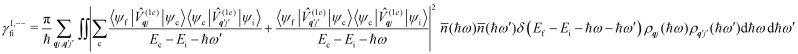
47
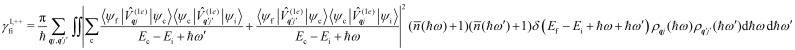
48
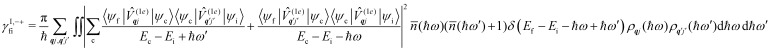
49
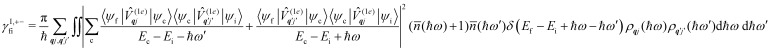


Note that the terms appearing in the double sums over ***q****j*,***q****j*′ in eqn (42)–(49) are symmetric with respect to exchange of the mode indices ***q****j* ↔ ***q***′*j*′. The terms involving simultaneous phonon emission and absorption (labelled by superscripts +− and −+) simply transform into each other when swapping mode indices. Therefore, the double summation can be replaced by twice the restricted sum over pairs of modes (***q****j* ≥ ***q***′*j*′) and a double-counting correction factor for the case in which the mode indices are the same 
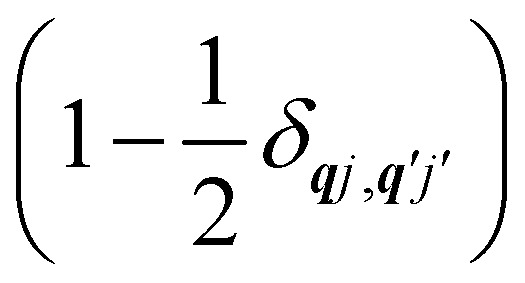
 inserted between the summation and double integral, for computational efficiency; we note that all terms should have the factor of 
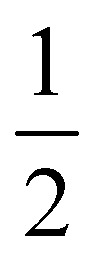
 and not some with 
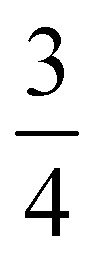
 as given in ref. 14.

With these transition rates calculated (*n.b.* total transition rates *γ*_fi_ are the sum of all contributions, eqn (40)–(49)), simulation of the spin dynamics is then a case of solving a classical rate equation for the electronic eigenstate populations:^63^50
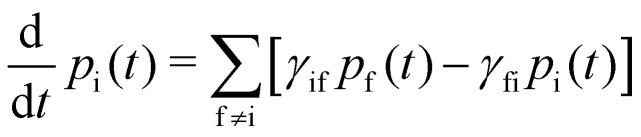
For this method to be valid, the γ_fi_ must obey the principle of detailed balance such that in equilibrium the forwards and backwards probability currents γ_fi_*p*_i_ and γ_if_*p*_f_ are equal due to microscopic reversibility:51
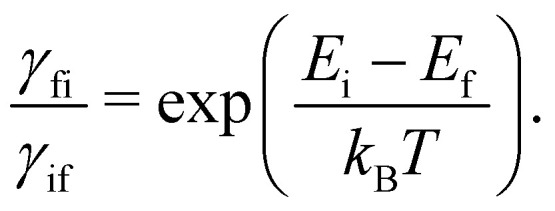


Solving the coupled differential eqn (50) can be achieved by constructing the matrix ***Γ*** with elements 
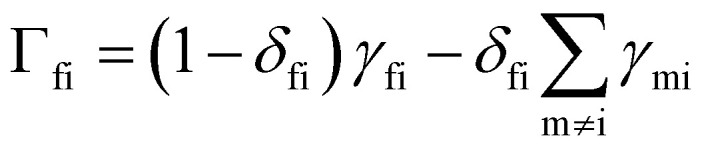
 (note that ***Γ*** here is the rate matrix and not the origin of the BZ, nor is it the phonon linewidth) and diagonalising‡‡Note that care must be taken with numerical diagonalisation of ***Γ*** where eigenvalues span many orders of magnitude and limitations due to finite numerical precision become important. For example, magnetic relaxation rates are usually on the order of 10^5^ s^−1^ to 10^−5^ s^−1^, while the “fast fluctuations” can be ∼10^13^ s^−1^, which sets the numerical value of “zero” as ∼10^−3^ s^−1^ in double precision arithmetic, which clearly poses a problem for calculation of rates at low temperature. However, using quadruple precision arithmetic, numerical “zero” would be ∼10^−21^ s^−1^ in the presence of eigenvalues of ∼10^13^ s^−1^. it (*i.e.* finding the matrix ***ϕ***, that transforms ***Γ*** into a diagonal form ***Λ*** = ***ϕ***^−1^***Γϕ***;).^63^ The eigenvalues of ***Γ*** (diagonal entries of ***Λ***) are a set of 2*J* + 1 characteristic rates −*τ*_k_^−1^, each corresponding to a so-called “normal mode” of relaxation (*n.b.* these are not the normal modes of vibration, nor are they different relaxation mechanisms). Of these normal modes, one rate corresponds to the system in equilibrium and is identically zero. When considering single-phonon processes alone for SMMs, often the Orbach mechanism dominates, and one of the normal modes of relaxation corresponds to the rate of this over-barrier process, which is many orders of magnitude slower than the remaining modes consisting of fast fluctuations of the populations of the states on either side of the barrier.^3^ Hence, often this rate is taken to be the same as the magnetic relaxation rate. In principle the spin dynamics of the system evolve under the influence of all normal modes of relaxation, so the magnetic relaxation rate should be determined by first simulating a magnetisation decay process and then fitting the trace with an exponential decay to give τ:52
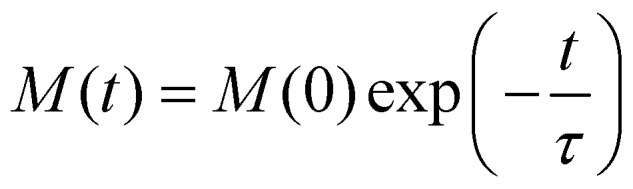
To do so, one must first calculate the population of each state |*ψ*_i_〉 as a function of time *t*:53
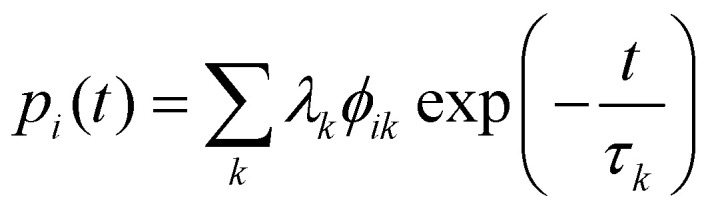
with ***λ*** = ***ϕ***^−1^**p**_0_, where **p**_0_ is a vector specifying the initial populations of the states; to simulate magnetic relaxation, one of the degenerate ground states would have unit population and all other states zero population. In this approach, the initial population evolves under the influence of each normal mode of relaxation (eigenvector of **Γ**). Then, the time-dependence of the total magnetisation is given as the sum over the magnetisation of each state, weighted by its population:54
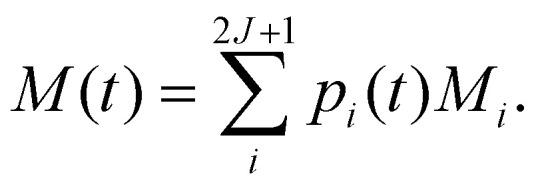


## 7 Practical considerations for *ab initio* calculation of spin–phonon coupling

Following the above theoretical descriptions, a practical *ab initio* calculation of spin–phonon coupling and spin dynamics requires four steps: (i) optimisation of the structure and calculation of the phonon modes; (ii) calculation of the equilibrium electronic structure; (iii) calculation of the spin–phonon coupling terms; and (iv) simulation of the spin dynamics.

The first step is usually performed using (semi-)local DFT methods, which are currently the only tractable methods for optimisation and lattice-dynamics calculations on periodic molecular crystals. Periodic DFT calculations commonly describe the valence electronic wavefunctions with a plane-wave basis set and use pseudopotentials to describe the nuclei and core electrons (*e.g.* in VASP,^64,65^ CASTEP^66^ or CP2K^67^), but all-electron calculations and atom-centred Gaussian-type atomic orbital basis sets are also an option (*e.g.* in CRYSTAL^68^). Optimisation of molecular crystal structures should generally be performed using a method including dispersion corrections (*e.g.* DFT-D^69^) in order to obtain qualitatively correct intermolecular potential energies.

Even when starting the optimisation of a molecular crystal from an experimental structure obtained with X-ray diffraction, the optimisation process is not necessarily straightforward owing to the presence of crystallographic disorder and shallow potential energy surfaces that can result in the optimisation algorithm getting stuck in a local maximum. The hallmark of a geometry optimisation landing in a local maximum on the PES is the presence of imaginary phonon modes (*ω*^2^ < 0) at the BZ centre (Γ point). These “dynamical instabilities” imply that a distortion of the atoms in the unit cell along the imaginary modes will lower the total energy, and hence that the unit cell contents are not fully optimised. Just like for molecular structure optimisations in the gas-phase, the imaginary modes can be removed by distorting the geometry along the eigenvector(s) corresponding to the imaginary phonon mode(s) and re-starting the optimisation at the minimum energy point along the distortion path. Doing so may result in a lowering of the crystallographic spacegroup symmetry. Typically one would distort along the largest magnitude imaginary mode, although this does not necessarily lead to the deepest energy minimum. After optimisation, the Γ-point phonon spectrum should be recalculated and checked for imaginary modes, and this process repeated until they are all removed.

When all phonon modes have *ω*^2^ ≥ 0 at the Γ point, then the unit cell can be considered optimised. Next, the phonon modes and their dispersion in reciprocal space should be calculated. For this, one chooses a path in reciprocal space visiting the unique high-symmetry ***q***-points in the BZ, which are determined by the crystallographic spacegroup. If all phonon branches have non-imaginary energies at all the high-symmetry wavevectors in the BZ, then the phonon calculation can be considered adequate. If there are imaginary modes at non-zero wavevectors, then these should be investigated. Off-Γ imaginary modes can arise in two cases: (i) interpolation artefacts for wavevectors that are not commensurate with the supercell expansion used in a finite-displacement calculation (or are not included in the “coarse” ***q***-point mesh in a perturbation-theory calculation); or (ii) “true” imaginary modes that indicate a dynamical instability for which an expanded unit-cell is required to contain the energy-lowering distortion. To check which case is causing the imaginary mode, one must perform the dispersion calculation with an expanded supercell (or a larger “coarse” mesh) that includes the wavevector(s) with imaginary mode(s). If the mode(s) becomes real, then it is case (i) and the phonons must simply be calculated in the larger supercell. If the mode remains imaginary, then it is a case of (ii) and the unit cell must be enlarged to contain the wavevector in question and the structure re-optimised. Checks for imaginary modes must then be repeated on the enlarged unit cell, and further calculations performed as necessary to remove them. In general, using a larger supercell expansion when calculating the phonon modes, which is equivalent to finer ***q***-point sampling, will improve the accuracy with which the frequencies of off-Γ modes are evaluated, but this is limited in practice by computational cost.

In the case of low-symmetry molecular crystals, it may not always be computationally tractable to remove all imaginary modes. As an example, we recently calculated the phonon spectra of [Dy(Cp^ttt^)_2_][B(C_6_F_5_)_4_] (Cp^ttt^ = 1,2,4-^*t*^Bu_3_-C_5_H_2_; ^*t*^Bu = C(CH_3_)_3_) at various pressures, and found that a 2 × 2 × 1 supercell with 1104 atoms was required to remove imaginary modes at most pressure points. Despite this, imaginary modes with *ħω* ∼ 10*i* cm^−1^ persisted at two pressures, and phonon calculations with the 2 × 2 × 2 supercell (2208 atoms) required to remove them were simply not computationally feasible.^70^ A detailed review of the physical significance of imaginary modes, possible causes, and treatments can be found in ref. 71.

This workflow for determining phonon spectra applies equally to all molecular crystals, however there are extra considerations for metal ions. For 4f elements, the near-degenerate, core-like 4f electrons, which present a problem for the single-determinant ansatz of DFT (see below) can be resolved by subsuming the 4f electrons into the pseudopotential core using “f-in-core” potentials – this is an acceptable approximation as the potential energy surface is largely unaffected by the 4f electrons. For d-block or 5f elements, the substantial bonding character of the d and 5f electrons makes this approach inappropriate, and hence these calculations are more challenging. Here, the self-interaction error in (semi-)local DFT functionals tends to overly delocalise the d- or f-states, which can be empirically corrected with an additional on-site term known as the Hubbard *U* parameter using the DFT+*U* method.^72,73^ However, even this approach does not guarantee success, and often simply converging the electronic wavefunction is a challenge and can make it difficult both to optimise the structure and, subsequently, to calculate the accurate forces required for phonon calculations. In these cases, sometimes substitution of the open-shell metal ion for an isovalent, closed-shell analogue with similar chemistry (*e.g.* replacing Co(ii) with Zn(ii) in octahedral environments to remove complications from the ground orbital triplet) is a suitable approximation.

The second step, calculation of the equilibrium electronic structure, requires explicit treatment of ground and excited electronic states, as well as inclusion of spin–orbit coupling, in order to accurately represent the magnetic spin states in molecules. Compared to closed-shell molecules, for which DFT is by far the dominant approach, such “single-determinant” electronic structure methods applied to open-shell molecules fail to correctly describe the ground state, where a single electron configuration fails as a qualitatively correct description of the electronic wavefunction.^74^ Instead, a multi-determinantal method is required to include the ground and excited electron configurations relevant for the magnetic properties. Often in the case of monometallic metal complexes, the magnetic states are well-localised and the d- or f-orbitals are the most important (this is obviously not true in the presence of strong magnetic coupling between multiple spin centres, *e.g.* for [Cp^^i^Pr_5_^DyI_3_DyCp^^i^Pr_5_^]^6^). Here, the complete active space self-consistent field (CASSCF)^75^ method provides a suitable approximation, where all electron configurations are included within a given “active” orbital space; this method is available in many codes (*e.g.* in OpenMolcas,^41,76^ Orca^77^ and Q-Chem^78^). In the case of Ln^3+^ complexes, the active space typically includes the seven 4f orbitals occupied by n electrons. The number of states and different spin multiplicities included in the calculation is user-determined, and for magnetic properties it is advised to be guided by the low-lying Russell–Saunders atomic terms ^2*S*+1^*L*.^22,35^ Spin–orbit coupling can then be added in a second step by mixing the CASSCF states of different spin multiplicities,^79^ leading to states representing the ^2*S*+1^*L*_*J*_ multiplets split into 2*J* + 1 components by the CF potential. Owing to the relatively small CF splitting of the 4f orbitals, the low-lying electronic states of Ln complexes can be well-described by a model crystal field Hamiltonian (eqn (13)), where the CFPs can be projected directly from the *ab initio* calculation.^80^ In general, any model Hamiltonian can be projected from such calculations for d- or 5f-containing molecules.^41^

While the CASSCF method provides an effective treatment of the so-called “static” electron correlation (arising from the multi-determinantal character of the electronic states), it does not do a good job of describing the “dynamic” electron correlation missing from the mean-field description of electron–electron repulsion (DFT has been so successful because it can approximate this second type of electron correlation very efficiently). This can be overcome by corrections on top of a CASSCF reference wave function either by using variational methods such as multi-reference configuration interaction (MRCI),^81^ or through the application of many-body perturbation theory, including methods such as second-order complete active space perturbation theory (CASPT2)^82,83^ and second-order *n*-electron valence state perturbation theory (NEVPT2),^84^ which often provide increased accuracy for magnetic properties.^80^

Correlated wave function methods such as CASSCF usually do not allow the explicit inclusion of extended environments (*i.e.* beyond the molecule of interest) due to their undesirable scaling behaviour with system size. As an alternative, environments can be included through implicit continuum models or at an atomistic level using hybrid approaches.^85^ We have recently demonstrated that an electrostatic potential derived from atomic point charges of environmental molecules^86^ can provide an appropriate description of the environment. In the case of crystalline environments, the electrostatic potential imposed by finite-size cluster models converges slowly towards the true Madelung potential of an infinite crystal, and has a non-trivial dependence on the shape of the point-charge cluster model.^87^ We have recently shown that the Madelung potential can be closely-approximated in a consistent manner by employing a spherical cluster of unit cells embedded in a conductor-like reaction field to screen the non-physical surface charges that arise from finite-size unit-cell expansions.^33^

**Fig. 7:**
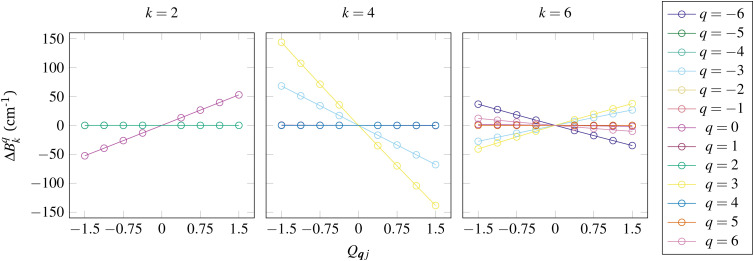
Change in each *B*^*q*^_*k*_ from the equilibrium values for [Yb(trensal)] as a function of mass- and frequency-weighted dimensionless displacement *Q*_***q****j*_ along a vibrational mode.^42^ Note that this mode is a totally-symmetric vibrational mode so that the *C*_3_ symmetry of [Yb(trensal)] is not broken and hence only certain *B*^*q*^_*k*_ are affected; hence, some are overlapping with zero magnitude.

The third step, computation of the spin–phonon coupling parameters, requires knowledge of the electronic response to nuclear distortions which is encoded in derivatives of the CFPs as presented in Section 5. The most obvious method to obtain these parameters is by computing the electronic structure and CFPs at distorted geometries along the normal mode coordinates and fitting the parameters to a polynomial (Fig. 7); such methods have been used by numerous authors.^16,31,38,88,89^ Clearly, however, for large and/or low symmetry molecules, many expensive *ab initio* calculations must be performed, and this is not yet even considering the second-order terms; to our knowledge only Sourav and Lunghi have attempted the latter, where they have used machine-learning methods to greatly simplify the calculation.^62,90^

Recently, we have shown that the spin–phonon coupling constants can alternatively be obtained analytically using a linear vibronic coupling (LVC) approximation^91–94^ based on a single *ab initio* calculation at the equilibrium geometry.^40^ The LVC method parametrises a first-order expansion of the CASSCF Hamiltonian matrix elements ***V*** in the atomic degrees of freedom *r*^*α*^_*κl*_ (for both the SMM and the environment):^95,96^

55 ***V*** = ***W***^(0)^ + ***W***^(1)^ + …

56 ***W***^(0)^ = diag(*ε*_0_,*ε*_1_,*ε*_2_,…)

57
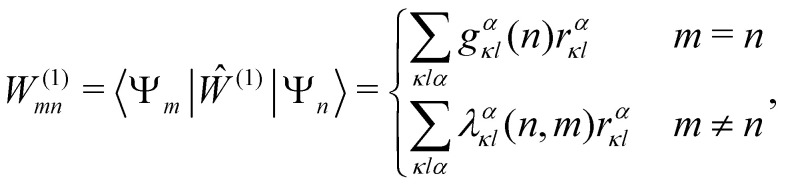
where ***W***^(*k*)^ collects terms of order *k* in the atomic coordinates, which are parameterised in case of *k* = 1 by electronic energies {*ε*_*n*_}, gradients {*g*^*α*^_*κl*_(*n*)} and non-adiabatic couplings (NACs) {*λ*^*α*^_*κl*_(*n*,*m*)} with respect to the CASSCF states *n*, *m*, which have recently become accessible for larger systems through the implementation of density fitting gradients in OpenMolcas.^92,97,98^ Although terms of *k* > 1 can be included in a straight-forward manner to give a higher-order extension of the method, the higher-order derivative couplings become increasingly expensive to compute, though we note that a scheme to parametrise a quadratic model from first-order derivatives has been reported.^99^ Furthermore, in cases where the inclusion of dynamical electron correlation is indispensable, the LVC model can be parameterised based on CASPT2 calculations.^100,101^

The final step is calculation of the spin dynamics and magnetic relaxation rates themselves. Here, one must either make their own implementation of the techniques described in Section 6 or rely on tools developed by others. We have developed a suite of Python tools (molcas_suite, angmom_suite and spin–phonon_suite, all available on the PyPI repository§§https://pypi.org/project/molcas-suite/, https://pypi.org/project/angmom-suite/, https://pypi.org/project/spin-phonon-suite/) to facilitate steps (i)–(iii) in conjunction with the VASP, phonopy and OpenMolcas codes, and the program Tau (available on GitLab¶¶https://gitlab.com/chilton-group/tau) to calculate magnetic relaxation rates; we have shown that this methodology gives quantitative accuracy with respect to experimental data.^33^ Lunghi and co-workers have developed their own methods in the MolForge package, available on GitHub.||||https://github.com/LunghiGroup/MolForge

## 8 Conclusions and outlook

Herein we have discussed the theory and practicalities of the calculation of phonon spectra, spin–phonon coupling and spin dynamics for molecular crystals, with a particular focus on magnetic relaxation in lanthanide single-molecule magnets. This area of research is at the cutting-edge of developments in electronic structure theory, quantum dynamics, machine learning and synthetic chemistry. The depth of knowledge required to take such calculations from start to finish is immense, and many works often make numerous assumptions on the background knowledge of the reader; we hope this Tutorial Review has lifted the veil on at least one aspect for the interested reader. We expect that as packages for these calculations become more advanced and automated, that exploration of chemical space with computational methods could start to make significant inroads into molecular design, both for improving timescales of classical memory storage in single-molecule magnets and coherence times for molecular qubits. Further, we expect that such methods will become crucial for understanding spin–phonon coupling and spin dynamics at the microscopic level for a broader class of nanomaterials in quantum science, such as spintronics and solid-state defect qubits, as well as in biological contexts such as enzyme catalysis and energy transfer.

## Author contributions

NFC proposed and supervised the work. All authors equally contributed to writing and editing the manuscript.

## Conflicts of interest

There are no conflicts to declare.

## Supplementary Material

CS-052-D2CS00705C-s001
