# Supplementary material for: Spin–phonon coupling and magnetic relaxation in single-molecule magnets
Source: Chem Soc Rev. 2023 Jun 28;52(14):4567–85. doi: 10.1039/d2cs00705c (PMC10351214; doi:10.1039/d2cs00705c)
Supplement: CS-052-D2CS00705C-s001 [file CS-052-D2CS00705C-s001.pdf]

Electronic Supplementary Material (ESI) for Chemical Society Reviews.
This journal is © The Royal Society of Chemistry 2023

Spin-phonon coupling and magnetic relaxation in single-molecule magnets: Supplementary information

Jon G. C. Kragskow,[†] Andrea Mattioni,[*,†] Jakob K. Staab,[†] Daniel Reta,[†,‡,¶,§] Jonathan M. Skelton,[*,†] and Nicholas F. Chilton[*,†]

†*Department of Chemistry, University of Manchester, Manchester M13 9PL, UK*

‡*Faculty of Chemistry, The University of the Basque Country UPV/EHU, 20018, Donostia, Spain*

¶*Donostia International Physics Center (DIPC), 20018, Donostia, Spain*

§*IKERBASQUE, Basque Foundation for Science, 48013, Bilbao, Spain*

E-mail: andrea.mattioni@manchester.ac.uk; jonathan.skelton@manchester.ac.uk; nicholas.chilton@manchester.ac.uk

Lattice-dynamics calculations

Lattice-dynamics calculations were performed using pseudopotential plane-wave density-functional theory (DFT), as implemented in the `Vienna Ab initio Simulation Package` (`VASP`) code,[1] in conjunction with the supercell finite-displacement approach implemented in the `Phonopy` code.[2]

Vibrational modes of CO_2

Calculations were performed on an isolated CO_2 molecule placed at the centre of a large cubic box, with an initial 15 Å between periodic images, using the PBE exchange-correlation functional[3] with the DFT-D3 dispersion correction (i.e. PBE-D3).[4] The ion cores were modelled using projector augmented-wave (PAW) pseudopotentials[5,6] with the C and O 2s/2p electrons in the valence region. The valence Kohn-Sham wavefunctions were represented in a plane-wave basis with an 800 eV kinetic-energy cutoff and sampled at the Γ point (i.e. a single $\boldsymbol{k}$-point). The atomic positions were optimised while keeping the cell shape and volume fixed, and the phonon frequencies and eigenvectors at the Brillouin-zone centre ($\boldsymbol{q} = \Gamma$) were computed using the finite-displacement routines in VASP with a displacement step of 10^{-2} Å. To ensure accurate forces, the PAW projection was performed in reciprocal space and an enhanced charge-density grid with 8× as many points as the standard grids was used to represent the augmentation charges.

Phonon dispersion and density of states of NaCl

Calculations were performed on the two-atom primitive cell of NaCl (spacegroup $Fm\bar{3}m$) using the PBEsol exchange-correlation functional.[7] The ion cores were modelled using PAW pseudopotentials with the Na 3s and Cl 3s/3p electrons in the valence region. The valence wavefunctions were described using a plane-wave basis with a 550 eV kinetic-energy cutoff and a Γ-centered Monkhorst-Pack $\boldsymbol{k}$-point grid[8] with 8×8×8 subdivisions. The cell volume

was fully optimised to tolerances of 10^{-8} eV on the electronic total energy and 10^{-2} eV $\mathring{A}^{-1}$ on the forces. The second-order interatomic force constants (IFCs) were determined in a 4 $\times$ 4 $\times$ 4 supercell of the primitive cell (128 atoms) using the default displacement step of 10^{-2} Å, and the $\boldsymbol{k}$-point sampling reduced accordingly. The PAW projection was performed in reciprocal space and an additional charge-density grid with 8$\times$ as many points as the standard grids was used to represent the augmentation charges. The atom-projected phonon density of states was obtained by interpolating the phonon frequenies and eigenvectors onto a uniform Γ-centered $\boldsymbol{q}$-point mesh with 48×48×48 subdivisions. The phonon dispersion was obtained by interpolating the frequencies along strings of $\boldsymbol{q}$-points passing through the $\boldsymbol{q} = L$, Γ and X high-symmetry wavevectors in the $Fm\bar{3}m$ Brillouin zone. Non-analytical corrections to the phonon dispersion to account for the splitting of the longitudinal and transverse modes close to $\boldsymbol{q} = \Gamma$ were included using the approach of Gonze *et al.*,[9,10] with the required high-frequency dielectric constant ε^{∞} and Born charge tensors $\boldsymbol{Z}^{*}$ determined using the density-functional perturbation theory (DFPT) routines in VASP.[11]

Phonon dispersion and density of states of crystalline NH_3

Calculations were performed on the cubic crystaline phase of NH_3 (spacegroup $P2_13$) using PBE-D3. The ion cores were modelled using PAW pesudopotentials with the H 1s and N 2s/2p electrons in the valence region. The valence electronic structure was modelled using a plane-wave basis with an 800 eV cutoff and a Γ-centered 2×2×2 $\boldsymbol{k}$-point grid. The initial structure was taken from the Materials Project database[12] (**mp-29145**) and has four NH_3 molecules (16 atoms) in the primitive cell. The atomic positions and unit-cell volume were optimised to tolerances of 10^{-8} eV on the total energy and 10^{-2} eV $\mathring{A}^{-1}$ on the forces. The second-order IFCs were determined in a 4×4×4 supercell with 1,024 atoms using a 10^{-2} Å displacement step, and the $\boldsymbol{k}$-point sampling reduced accordingly. The PAW projection was performed in reciprocal space and an enhanced charge-density grid with 8× as many points as the standard grids was used to represent the augmentation charges. The phonon density

of states was evaluated on a $24 \times 24 \times 24$ $\boldsymbol{q}$-point grid, and the dispersion wa computed along a path including the high-symmetry X, Γ, M and R wavevectors in the $P2_13$ Brillouin zone. In order to classify the phonon modes, the modes at $\boldsymbol{q} = \Gamma$ were visualised using the `MolecularCrystalPhononAnimation` code.[13]

Spin-phonon coupling formalism

As noted in the main text, the phonon position operators

$$\hat{Q}_{\boldsymbol{q}j} = \sqrt{\frac{\hbar}{2\omega_{\boldsymbol{q}j}}} \left(\hat{a}_{\boldsymbol{q}j} + \hat{a}^{\dagger}_{-\boldsymbol{q}j} \right) \tag{1}$$

are in general non-Hermitian which complicates the further derivation and practical implementation of the rate expressions, i.e. loss of the Hermitian property of each individual terms in Equation 16 in the main text, and complex-valued atomic displacements. In particular, $\hat{Q}_{\boldsymbol{q}j}$ operators of Γ and zone boundary q-points with $q_x, q_y, q_z \in \{0, 0.5\}$ are Hermitian while it is only at intermediate q-points denoted by the set $\boldsymbol{q}^*$ where we choose to introduce a re-grouping of spin-phonon coupling operator terms as shown in Equation 2. The original normal mode operators are partitioned into Hermitian and anti-Hermitian parts and pairs of terms representing conjugate q-points (using the relation $\hat{Q}^{\dagger}_{\boldsymbol{q}j} = \hat{Q}_{-\boldsymbol{q}j}$) are grouped together, which restricts the summation over half of the Brillouin zone denoted by the set $\boldsymbol{q}^+$.

$$
\begin{aligned}
\sum_{\substack{\boldsymbol{q}\in\boldsymbol{q}^* \\ j}} \hat{V}^{(1)}_{\boldsymbol{q}j} &= \sum_{\substack{\boldsymbol{q}\in\boldsymbol{q}^* \\ j}} \frac{\partial \hat{H}_{\mathrm{S}}}{\partial Q_{\boldsymbol{q}j}} \otimes \widehat{Q}_{\boldsymbol{q}j} \\
&= \sum_{\substack{\boldsymbol{q}\in\boldsymbol{q}^* \\ j}} \frac{\partial \hat{H}_{\mathrm{S}}}{\partial Q_{\boldsymbol{q}j}} \otimes \underbrace{\frac{1}{2}\left(\widehat{Q}_{\boldsymbol{q}j} + \widehat{Q}^{\dagger}_{\boldsymbol{q}j}\right)}_{\text{Hermitian}} + \underbrace{\frac{1}{2}\left(\widehat{Q}_{\boldsymbol{q}j} - \widehat{Q}^{\dagger}_{\boldsymbol{q}j}\right)}_{\text{anti-Hermitian}} \\
&= \sum_{\substack{\boldsymbol{q}\in\boldsymbol{q}^+ \\ j}} \left(\frac{\partial \hat{H}_{\mathrm{S}}}{\partial Q_{\boldsymbol{q}j}} + \frac{\partial \hat{H}_{\mathrm{S}}}{\partial Q_{-\boldsymbol{q}j}}\right) \otimes \frac{1}{2}\left(\widehat{Q}_{\boldsymbol{q}j} + \widehat{Q}_{-\boldsymbol{q}j}\right) \\
&+ \sum_{\substack{\boldsymbol{q}\in\boldsymbol{q}^+ \\ j}} i\left(\frac{\partial \hat{H}_{\mathrm{S}}}{\partial Q_{\boldsymbol{q}j}} - \frac{\partial \hat{H}_{\mathrm{S}}}{\partial Q_{-\boldsymbol{q}j}}\right) \otimes \frac{1}{2i}\left(\widehat{Q}_{\boldsymbol{q}j} - \widehat{Q}_{-\boldsymbol{q}j}\right) \\
&= \sum_{\substack{\boldsymbol{q}\in\boldsymbol{q}^+ \\ j}} \left(\frac{\partial \hat{H}_{\mathrm{S}}}{\partial Q_{\boldsymbol{q}j}} + \frac{\partial \hat{H}_{\mathrm{S}}}{\partial Q_{-\boldsymbol{q}j}}\right) \otimes \frac{1}{2}\sqrt{\frac{\hbar}{2\omega_{\boldsymbol{q}j}}}\left(\hat{a}_{\boldsymbol{q}j} + \hat{a}^{\dagger}_{-\boldsymbol{q}j} + \hat{a}_{-\boldsymbol{q}j} + \hat{a}^{\dagger}_{\boldsymbol{q}j}\right) \\
&+ \sum_{\substack{\boldsymbol{q}\in\boldsymbol{q}^+ \\ j}} i\left(\frac{\partial \hat{H}_{\mathrm{S}}}{\partial Q_{\boldsymbol{q}j}} - \frac{\partial \hat{H}_{\mathrm{S}}}{\partial Q_{-\boldsymbol{q}j}}\right) \otimes \frac{1}{2i}\sqrt{\frac{\hbar}{2\omega_{\boldsymbol{q}j}}}\left(\hat{a}_{\boldsymbol{q}j} + \hat{a}^{\dagger}_{-\boldsymbol{q}j} - \hat{a}_{-\boldsymbol{q}j} - \hat{a}^{\dagger}_{\boldsymbol{q}j}\right) \\
&= \sum_{\substack{\boldsymbol{q}\in\boldsymbol{q}^+ \\ j}} \frac{1}{2}\sqrt{\frac{\hbar}{\omega_{\boldsymbol{q}j}}}\left(\frac{\partial \hat{H}_{\mathrm{S}}}{\partial Q_{\boldsymbol{q}j}} + \frac{\partial \hat{H}_{\mathrm{S}}}{\partial Q_{-\boldsymbol{q}j}}\right) \otimes \left(\frac{\hat{a}_{\boldsymbol{q}j} + \hat{a}_{-\boldsymbol{q}j}}{\sqrt{2}} + \frac{\hat{a}^{\dagger}_{\boldsymbol{q}j} + \hat{a}^{\dagger}_{-\boldsymbol{q}j}}{\sqrt{2}}\right) \\
&+ \sum_{\substack{\boldsymbol{q}\in\boldsymbol{q}^+ \\ j}} \frac{i}{2}\sqrt{\frac{\hbar}{\omega_{\boldsymbol{q}j}}}\left(\frac{\partial \hat{H}_{\mathrm{S}}}{\partial Q_{\boldsymbol{q}j}} - \frac{\partial \hat{H}_{\mathrm{S}}}{\partial Q_{-\boldsymbol{q}j}}\right) \otimes \left(\frac{\hat{a}_{\boldsymbol{q}j} - \hat{a}_{-\boldsymbol{q}j}}{\sqrt{2}i} - \frac{\hat{a}^{\dagger}_{\boldsymbol{q}j} - \hat{a}^{\dagger}_{-\boldsymbol{q}j}}{\sqrt{2}i}\right) \\
&= \sum_{\substack{\boldsymbol{q}\in\boldsymbol{q}^* \\ j}} \frac{\partial \hat{H}_{\mathrm{S}}}{\partial X_{\boldsymbol{q}j}} \otimes \left(\hat{b}_{\boldsymbol{q}j} + \hat{b}^{\dagger}_{\boldsymbol{q}j}\right). \qquad (2)
\end{aligned}
$$

In the last line we have introduced a new set of phonon creation operators

$$
\hat{b}_{\boldsymbol{q}j} = \begin{cases} \frac{\hat{a}_{\boldsymbol{q}j} + \hat{a}_{-\boldsymbol{q}j}}{\sqrt{2}} & \text{if } \boldsymbol{q} \in \boldsymbol{q}^+, \\ \frac{\hat{a}_{-\boldsymbol{q}j} - \hat{a}_{\boldsymbol{q}j}}{\sqrt{2}i} & \text{if } -\boldsymbol{q} \in \boldsymbol{q}^+, \end{cases} \qquad (3)
$$

which are defined for $\boldsymbol{q} \in \boldsymbol{q}^*$, and the Hermitean mode position operators $\hat{X}_{\boldsymbol{q}j} = \hat{b}_{\boldsymbol{q}j} + \hat{b}^{\dagger}_{\boldsymbol{q}j}$.

The spin-phonon couplings $\partial \hat{H}_{\mathrm{S}}/\partial X_{\boldsymbol{q}j}$ can be calculated in terms of the cartesian dis-

placements as

$$\begin{aligned}\frac{\partial \hat{H}_{\rm S}}{\partial X_{\boldsymbol{q}j}} &= \frac{1}{2}\sqrt{\frac{\hbar}{\omega_{\boldsymbol{q}j}}}\left(\frac{\partial \hat{H}_{\rm S}}{\partial Q_{\boldsymbol{q}j}}+\frac{\partial \hat{H}_{\rm S}}{\partial Q_{-\boldsymbol{q}j}}\right)\\ &= \frac{1}{2}\sqrt{\frac{\hbar}{\omega_{\boldsymbol{q}j}}}\sum_{\kappa l\alpha}\frac{\partial \hat{H}_{\rm S}}{\partial r^{\alpha}_{\kappa l}}\left(\frac{\partial r^{\alpha}_{\kappa l}}{\partial Q_{\boldsymbol{q}j}}+\frac{\partial r^{\alpha}_{\kappa l}}{\partial Q_{-\boldsymbol{q}j}}\right)\\ &= \sum_{\kappa l\alpha}\frac{\partial \hat{H}_{\rm s}}{\partial r^{\alpha}_{\kappa l}}\sqrt{\frac{\hbar}{N m_{\kappa}\omega_{\boldsymbol{q}j}}}{\rm Re}\left[W^{\alpha}_{\kappa}(\boldsymbol{q}j)\exp\left(i\boldsymbol{q}\cdot\boldsymbol{r}_{\kappa l}\right)\right]\end{aligned} \tag{4}$$

if $\boldsymbol{q}\in\boldsymbol{q}^{+}$, and

$$\begin{aligned}\frac{\partial \hat{H}_{\rm S}}{\partial X_{\boldsymbol{q}j}} &= \frac{1}{2i}\sqrt{\frac{\hbar}{\omega_{\boldsymbol{q}j}}}\left(\frac{\partial \hat{H}_{\rm S}}{\partial Q_{\boldsymbol{q}j}}-\frac{\partial \hat{H}_{\rm S}}{\partial Q_{-\boldsymbol{q}j}}\right)\\ &= \frac{1}{2i}\sqrt{\frac{\hbar}{\omega_{\boldsymbol{q}j}}}\sum_{\kappa l\alpha}\frac{\partial \hat{H}_{\rm S}}{\partial r^{\alpha}_{\kappa l}}\left(\frac{\partial r^{\alpha}_{\kappa l}}{\partial Q_{\boldsymbol{q}j}}-\frac{\partial r^{\alpha}_{\kappa l}}{\partial Q_{-\boldsymbol{q}j}}\right)\\ &= \sum_{\kappa l\alpha}\frac{\partial \hat{H}_{\rm s}}{\partial r^{\alpha}_{\kappa l}}\sqrt{\frac{\hbar}{N m_{\kappa}\omega_{\boldsymbol{q}j}}}{\rm Im}\left[W^{\alpha}_{\kappa}(\boldsymbol{q}j)\exp\left(i\boldsymbol{q}\cdot\boldsymbol{r}_{\kappa l}\right)\right]\end{aligned} \tag{5}$$

if $-\boldsymbol{q}\in\boldsymbol{q}^{+}$, where we have used $W^{\alpha}_{\kappa}(-\boldsymbol{q}j)=W^{\alpha}_{\kappa}(\boldsymbol{q}j)^{*}$.

The Γ point and the q-points at the edge of the Brillouin zone need to be handled separately. In that case, $\widehat{Q}_{\boldsymbol{q}j}$ is already Hermitean, and we define $\hat{X}_{\boldsymbol{q}j}=\sqrt{\frac{2\omega_{\boldsymbol{q}j}}{\hbar}}\widehat{Q}_{\boldsymbol{q}j}=\hat{a}_{\boldsymbol{q}j}+\hat{a}^{\dagger}_{\boldsymbol{q}j}$, such that the spin-phonon coupling operator simply becomes

$$\frac{\partial \hat{H}_{\rm S}}{\partial X_{\boldsymbol{q}j}}=\sqrt{\frac{\hbar}{2\omega_{\boldsymbol{q}j}}}\frac{\partial \hat{H}_{\rm S}}{\partial Q_{\boldsymbol{q}j}}=\frac{1}{\sqrt{2}}\sum_{\kappa l\alpha}\frac{\partial \hat{H}_{\rm s}}{\partial r^{\alpha}_{\kappa l}}\sqrt{\frac{\hbar}{N m_{\kappa}\omega_{\boldsymbol{q}j}}}W^{\alpha}_{\kappa}(\boldsymbol{q}j)\exp\left(i\boldsymbol{q}\cdot\boldsymbol{r}_{\kappa l}\right) \tag{6}$$

if $\boldsymbol{q}\notin\boldsymbol{q}^{*}$. Note that for the practical implementation of Equation 6 we adjust the arbitrary complex phase of the eigenvector $\boldsymbol{W}(\boldsymbol{q}j)$ to obtain an overall real quantity for the displacement amplitudes $W^{\alpha}_{\kappa}(\boldsymbol{q}j)\exp\left(i\boldsymbol{q}\cdot\boldsymbol{r}_{\kappa l}\right)$. In analogy with the re-definitions above, second order coupling parameters $\partial^{2}\hat{H}_{\rm S}\Big/\partial X_{\boldsymbol{q}j}\,\partial X_{\boldsymbol{q}'j'}$ can be expressed in the same basis of normal mode operators $\hat{X}_{\boldsymbol{q}j}$.

Since the transformation defined in Equation (3) between the set of mode operators $\hat{a}_{\boldsymbol{q}j}$

and $\hat{b}_{\boldsymbol{q}j}$ is unitary, the canonical commutation relations are preserved, e.g. $[\hat{b}_{\boldsymbol{k}i}, \hat{b}^\dagger_{\boldsymbol{q}j}] = \delta_{ij}\delta_{\boldsymbol{qk}}$. This allows us to easily calculate the bath correlation functions that we will use to write down the quantum master equations for the reduced density matrix of the spin system in the next section. In particular, the two-time bath correlation function at thermal equilibrium, which determines the one-phonon (Orbach) rates, is given by

$$\langle \hat{X}_{\boldsymbol{q}j}(t)\hat{X}_{\boldsymbol{q}'j'}(t')\rangle_{\rm eq} = \delta_{\boldsymbol{qq}'}\delta_{jj'}\left[(\bar{n}_{\boldsymbol{q}j}+1)e^{-i\omega_{\boldsymbol{q}j}(t-t')} + \bar{n}_{\boldsymbol{q}j}e^{i\omega_{\boldsymbol{q}j}(t-t')}\right] =: \delta_{\boldsymbol{qq}'}\delta_{jj'}c_{\boldsymbol{q}j}(t-t'), \quad (7)$$

where $\bar{n}_{\boldsymbol{q}j} = 1/(e^{\hbar\omega_{\boldsymbol{q}j}/k_{\rm B}T}-1)$ is the Bose-Einstein occupation number. It can be shown that the four-time equilibrium correlation functions entering the Raman-I and Raman-II rates can be decomposed in terms of two-time correlations functions as

$$\begin{aligned}\langle \hat{X}_{\boldsymbol{q}_0j_0}(t_0)\hat{X}_{\boldsymbol{q}_1j_1}(t_1)\hat{X}_{\boldsymbol{q}_2j_2}(t_2)\hat{X}_{\boldsymbol{q}_3j_3}(t_3)\rangle_{\rm eq} &= \langle \hat{X}_{\boldsymbol{q}_0j_0}(t_0)\hat{X}_{\boldsymbol{q}_3j_3}(t_3)\rangle_{\rm eq}\langle \hat{X}_{\boldsymbol{q}_1j_1}(t_1)\hat{X}_{\boldsymbol{q}_2j_2}(t_2)\rangle_{\rm eq} \\ &+ \langle \hat{X}_{\boldsymbol{q}_0j_0}(t_0)\hat{X}_{\boldsymbol{q}_2j_2}(t_2)\rangle_{\rm eq}\langle \hat{X}_{\boldsymbol{q}_1j_1}(t_1)\hat{X}_{\boldsymbol{q}_3j_3}(t_3)\rangle_{\rm eq} \\ &+ \langle \hat{X}_{\boldsymbol{q}_0j_0}(t_0)\hat{X}_{\boldsymbol{q}_1j_1}(t_1)\rangle_{\rm eq}\langle \hat{X}_{\boldsymbol{q}_2j_2}(t_2)\hat{X}_{\boldsymbol{q}_3j_3}(t_3).\rangle_{\rm eq}\end{aligned} \quad (8)$$

This fact only relies on the operators $\hat{X}_{\boldsymbol{q}j}$ being Hermitian linear combinations of creation/annihilation operators, and on the Gaussian property of thermal states.

Quantum Master Equations

In this section we apply the time-convolutionless (TCL) expansion[14] of the generator of the reduced dynamics to obtain 2$^{\rm nd}$ and 4$^{\rm th}$ order quantum master equations describing the dynamics of a spin system coupled to a very large vibrational bath in thermal equilibrium. We consider both linear and quadratic couplings to a harmonic environment, in order to obtain expressions for the Orbach, Raman-I and Raman-II magnetic relaxation rates. The

system-bath coupling Hamiltonian is thus

$$\hat{H}_{\rm SB} = \sum_i \hat{V}_i \otimes \hat{X}_i + \frac{1}{2}\sum_{ij} \hat{W}_{ij} \otimes \hat{X}_i \hat{X}_j, \tag{9}$$

where $\hat{V}_i = \left(\frac{\partial \hat{H}_{\rm S}}{\partial X_i}\right)_{\rm eq}$ and $\hat{W}_{ij} = \left(\frac{\partial^2 \hat{H}_{\rm S}}{\partial X_i \partial X_j}\right)_{\rm eq}$ are the first and second derivatives of the spin Hamiltonian $\hat{H}_{\rm S}$ with respect to modes i and j at the equilibrium geometry. For ease of notation, we set $\hbar = 1$ and we drop the q-point dependence of the modes, letting a single mode index i run over both phonon bands and q-points within the first Brillouin zone. We also assume, without loss of generality, that every single term in the sums in Equation (9) is Hermitian, based on the results shown in the previous section.

Up to $4^{\rm th}$ order in the system-bath coupling, the spin reduced density matrix ρ evolves according to the quantum master equation

$$\frac{{\rm d}\rho}{{\rm d}t} = -i[\hat{H}_{\rm S}, \rho] + \mathcal{K}_2\rho + \mathcal{K}_4\rho. \tag{10}$$

The $4^{\rm th}$ order term consists of two contributions $\mathcal{K}_4 = \mathcal{K}_4^I + \mathcal{K}_4^{II}$, describing the effect of the linear spin-phonon coupling to $4^{\rm th}$ order ($\mathcal{K}_4^I$) and the $2^{\rm nd}$ order contribution of the quadratic spin-phonon coupling ($\mathcal{K}_4^{II}$).

The matrix element of the generator $\mathcal{K}_{ab,cd}$ describes the influence of $\langle\psi_c|\,\rho\,|\psi_d\rangle$ on the time evolution of $\langle\psi_a|\,\rho\,|\psi_b\rangle$. If we are only interested in relaxation rates for spin populations, we can focus just on the terms $\mathcal{K}_{ff,ii}$, which represents the transition rate between two eigenstates of the system Hamiltonian $H_{\rm S}$ at energies E_i and E_f. In general, population dynamics is influenced by the presence of coherences, i.e. $\mathcal{K}_{ff,ab} \neq 0$ for $a \neq b$. However, one often invokes the *secular approximation*[14] (or *rotating wave approximation*), which leads to a decoupling of populations from coherences, provided the system has a non-degenerate spectrum (in this context, "non-degenerate" implies $\omega_{if} \gg 2\pi\tau^{-1}$, which is easily satisfied for magnetic molecules in small magnetic fields of a few Oe). If the spectrum is degenerate,

it can be shown that the only coherence-to-population terms $\mathcal{K}_{ff,ab}$ surviving the secular approximation are the ones involving coherences between degenerate states, i.e. $E_a = E_b$. The secular approximation amounts to neglecting oscillatory terms in the interaction picture representation of the generator $\mathcal{K}$, based on the idea that energy gaps between system eigenstates correspond to frequencies that are much faster than the relaxation dynamics, and therefore average to zero on the long timescale of magnetic relaxation.

Orbach rates

To lowest order in the spin-phonon coupling, the rate of change of the spin reduced density matrix is given by the 2nd order TCL expansion (TCL2) in the linear system-bath coupling, which we call $\mathcal{K}_2\rho$. In the interaction picture, where $\tilde{\rho}(t) = e^{i\hat{H}_{\text{S}}t}\rho(t)e^{-i\hat{H}_{\text{S}}t}$, this is given by

$$\frac{\mathrm{d}\tilde{\rho}(t)}{\mathrm{d}t} = \tilde{\mathcal{K}}_2\tilde{\rho}(t) = -\sum_i \int_0^t \mathrm{d}t_1 c_i(t-t_1)[\hat{V}_i(t), \hat{V}_i(t_1)\tilde{\rho}(t)] + \text{ h.c.} \tag{11}$$

(h.c. means Hermitian conjugate) which is widely known as the *Redfield Equation.* The function $c_i(t-t_1)$ represents the two-time bath correlation function calculated on a thermal equilibrium state, i.e.

$$\langle \hat{X}_i(t)\hat{X}_j(t')\rangle_{\text{eq}} = \delta_{ij}c_i(t-t') = \delta_{ij}\left[e^{-i\omega_i(t-t')}(\bar{n}_i+1) + e^{i\omega_i(t-t')}\bar{n}_i\right] \tag{12}$$

where ω_i is the mode frequency and $\bar{n}_i = 1/(e^{\omega_i/k_{\text{B}}T}-1)$ is the occupation number at thermal equilibrium.

We introduce the jump operators between system energy levels

$$\hat{V}_i(\omega) = \sum_{E_a-E_b=\omega} |\psi_b\rangle\langle\psi_b|\,\hat{V}_i\,|\psi_a\rangle\langle\psi_a|\,, \tag{13}$$

which allow us to decompose the time evolution of system operators as

$$\hat{V}_i(t) = e^{i\hat{H}_{\mathrm{S}}t}\hat{V}_i e^{-i\hat{H}_{\mathrm{S}}t} = \sum_{\omega} e^{-i\omega t}\hat{V}_i(\omega). \tag{14}$$

This decomposition allows us to separate all the time-dependent factors in Equation (11) and perform the time integral. Transforming back to the Schrödinger picture, we obtain

$$\mathcal{K}_2\rho(t) = -\sum_i \sum_{\omega_1} R_i(\omega_1)[\hat{V}_i, \hat{V}_i(\omega_1)\rho(t)] + \text{ h.c.} \tag{15}$$

where the rate $R_i(\omega_1)$ is given by

$$R_i(\omega_1) = \mathrm{Re}\lim_{t\to\infty}\int_0^t \mathrm{d}t_1 e^{i\omega_1(t-t_1)}c_i(t-t_1). \tag{16}$$

Note that we have introduced two approximations. First, we have taken the upper limit of integration to go to infinity, i.e. $t \to \infty$. This corresponds to only focusing on the long-time limit of the Markovian dynamics. Second, we are deliberately neglecting the imaginary part of the rates. This is because we are only interested in the dissipative relaxation dynamics induced by the real part, as opposed to the oscillatory dynamics described by the imaginary part. The limit $t \to \infty$ gives rise to selection rules for transitions between spin states depending on the phonon energies, based on the relation

$$\lim_{t\to\infty}\frac{\sin(\omega t)}{\pi\omega} = \delta(\omega). \tag{17}$$

Substituting Equation (12) into the expression for $R_i(\omega_1)$, and calculating the contribution to $\langle\psi_f|\,\mathcal{K}_2\rho\,|\psi_f\rangle$ stemming from $\langle\psi_i|\,\rho\,|\psi_i\rangle$, we obtain the Orbach relaxation rates

$$(\mathcal{K}_2)_{ff,ii} = 2\pi\sum_i \left|V_i^{fi}\right|^2 \left[(\bar{n}_i + 1)\delta(\omega_{if} - \omega_i) + \bar{n}_i\delta(\omega_{if} + \omega_i)\right], \tag{18}$$

where we introduced the notation $V_i^{ab} = \langle\psi_a|\,\hat{V}_i\,|\psi_b\rangle$ and $\omega_{ab} = E_a - E_b$. Extension to

modes with finite linewidth is straightforward, and simply amounts to integrating the rate expression over a continuous distribution of mode energies described by a lineshape function (see main text).

Raman-II rates

The Raman-II relaxation mechanism is captured by the TCL2 expansion in the quadratic spin-phonon coupling in Equation (9). In this case, we need to account for the non-vanishing expectation value of the quadratic spin-phonon coupling at equilibrium, i.e. $\langle \hat{X}_i \hat{X}_j \rangle_{\text{eq}} = \delta_{ij} c_i(0) \neq 0$. We do so by adding and subtracting this expectation value to the bath operators and incorporating it into a redefined system Hamiltonian $\hat{H}_{\text{S}} + \frac{1}{2} \sum_{ij} \hat{W}_{ij} \langle \hat{X}_i \hat{X}_j \rangle_{\text{eq}}$. We rewrite the residual quadratic spin-phonon coupling as

$$\frac{1}{2} \sum_{ij} \hat{W}_{ij} \otimes \left(\hat{X}_i \hat{X}_j - \langle \hat{X}_i \hat{X}_j \rangle_{\text{eq}} \right) = \sum_{\mu} \hat{\mathcal{V}}_{\mu} \otimes \hat{\mathcal{X}}_{\mu}, \tag{19}$$

where the index μ runs over all mode pairs (i, j) (including $i = j$) and we have introduced $\hat{\mathcal{V}}_{\mu} = \hat{W}_{ij}/2$ and $\hat{\mathcal{X}}_{\mu} = \hat{X}_i \hat{X}_j - \langle \hat{X}_i \hat{X}_j \rangle_{\text{eq}}$. These definitions allow us to exploit the analogy with the TCL2 expansion for the linear coupling carried out in the previous section. Replacing $\hat{V}_i \to \hat{\mathcal{V}}_{\mu}$ in Equation (15) and $\hat{X}_i \to \hat{\mathcal{X}}_{\mu}$ in Equation (12), yields the Raman-II rates.

The two-time correlation function for the operators $\hat{\mathcal{X}}_{\mu}$ can be readily obtained from Equation (8), yielding

$$\langle \hat{\mathcal{X}}_{ij}(\tau) \hat{\mathcal{X}}_{kl} \rangle_{\text{eq}} = (\delta_{ik}\delta_{jl} + \delta_{il}\delta_{jk}) c_i(\tau) c_j(\tau). \tag{20}$$

Following the same steps outlined in the previous section for Orbach rates, we obtain the

Raman-II rates

$$(\mathcal{K}_4^{II})_{ff,ii} = \pi \sum_{ij} \left| W_{ij}^{fi} \right|^2 \Big[\delta(\omega_{if} - \omega_i - \omega_j) \ (\bar{n}_i + 1) \ (\bar{n}_j + 1) \\ + \delta(\omega_{if} - \omega_i + \omega_j) \ (\bar{n}_i + 1) \ \bar{n}_j \\ + \delta(\omega_{if} + \omega_i - \omega_j) \ \bar{n}_i \ (\bar{n}_j + 1) \\ + \delta(\omega_{if} + \omega_i + \omega_j) \ \bar{n}_i \ \bar{n}_j \Big]. \tag{21}$$

Raman-I rates

Following Breuer and Petruccione,[14] we write the TCL4 generator of the system reduced dynamics as

$$\tilde{\mathcal{K}}_4^I \tilde{\rho} = \sum_{ij} \int_0^t \mathrm{d}t_1 \int_0^{t_1} \mathrm{d}t_2 \int_0^{t_2} \mathrm{d}t_3 \Big\{ c_i(t-t_2) c_j(t_1-t_3) [\hat{V}_i(t), [\hat{V}_j(t_1), \hat{V}_i(t_2)] \hat{V}_j(t_3) \tilde{\rho}] \\ - c_i^*(t-t_2) c_j(t_1-t_3) [\hat{V}_i(t), \hat{V}_j(t_3) \tilde{\rho} [\hat{V}_j(t_1), \hat{V}_i(t_2)]] \\ + c_i(t-t_3) c_j(t_1-t_2) [\hat{V}_i(t), [\hat{V}_j(t_1), \hat{V}_i(t_3)] \hat{V}_j(t_2) \tilde{\rho}] \\ - c_i^*(t-t_3) c_j(t_1-t_2) [\hat{V}_i(t), \hat{V}_j(t_2) \tilde{\rho} [\hat{V}_j(t_1), \hat{V}_i(t_3)]] \\ + c_i(t-t_3) c_j(t_1-t_2) [\hat{V}_i(t), \hat{V}_j(t_1) [\hat{V}_j(t_2), \hat{V}_i(t_3)] \tilde{\rho}] \\ - c_i(t-t_3) c_j(t_1-t_2) [\hat{V}_i(t), [\hat{V}_j(t_2), \hat{V}_i(t_3)] \tilde{\rho} \hat{V}_j(t_1)] \Big\} \\ + \text{ h.c.} \tag{22}$$

where the tilde indicates that we are in the interaction picture. Using the same spectral decomposition of $\hat{V}_i(t)$ introduced above, we write the Schrödinger picture representation of

the TCL4 generator as

$$
\begin{aligned}
\mathcal{K}_4^I \rho = \sum_{\omega_1,\omega_2,\omega_3} \sum_{ij} \Big\{ & R_{ij}^{(1)}(\omega_1,\omega_2,\omega_3)[\hat{V}_i, [\hat{V}_j(\omega_1), \hat{V}_i(\omega_2)]\hat{V}_j(\omega_3)\rho] \\
& - R_{ij}^{(2)}(\omega_1,\omega_2,\omega_3)[\hat{V}_i, \hat{V}_j(\omega_3)\rho[\hat{V}_j(\omega_1), \hat{V}_i(\omega_2)]] \\
& + R_{ij}^{(3)}(\omega_1,\omega_2,\omega_3)[\hat{V}_i, [\hat{V}_j(\omega_1), \hat{V}_i(\omega_3)]\hat{V}_j(\omega_2)\rho] \\
& - R_{ij}^{(4)}(\omega_1,\omega_2,\omega_3)[\hat{V}_i, \hat{V}_j(\omega_2)\rho[\hat{V}_j(\omega_1), \hat{V}_i(\omega_3)]] \\
& + R_{ij}^{(3)}(\omega_1,\omega_2,\omega_3)[\hat{V}_i, \hat{V}_j(\omega_1)[\hat{V}_j(\omega_2), \hat{V}_i(\omega_3)]\rho] \\
& - R_{ij}^{(3)}(\omega_1,\omega_2,\omega_3)[\hat{V}_i, [\hat{V}_j(\omega_2), \hat{V}_i(\omega_3)]\rho\hat{V}_j(\omega_1)] \Big\} \\
& + \text{ h.c.}
\end{aligned} \tag{23}
$$

where we have defined

$$
R_{ij}^{(1)}(\omega_1,\omega_2,\omega_3) = \mathrm{Re}\lim_{t\to\infty}\int_0^t \mathrm{d}t_1 \int_0^{t_1} \mathrm{d}t_2 \int_0^{t_2} \mathrm{d}t_3 e^{-i(\omega_1 t_1+\omega_2 t_2+\omega_3 t_3)} e^{i(\omega_1+\omega_2+\omega_3)t} c_i(t-t_2)c_j(t_1-t_3) \tag{24}
$$

$$
R_{ij}^{(2)}(\omega_1,\omega_2,\omega_3) = \mathrm{Re}\lim_{t\to\infty}\int_0^t \mathrm{d}t_1 \int_0^{t_1} \mathrm{d}t_2 \int_0^{t_2} \mathrm{d}t_3 e^{-i(\omega_1 t_1+\omega_2 t_2+\omega_3 t_3)} e^{i(\omega_1+\omega_2+\omega_3)t} c_i^*(t-t_2)c_j(t_1-t_3) \tag{25}
$$

$$
R_{ij}^{(3)}(\omega_1,\omega_2,\omega_3) = \mathrm{Re}\lim_{t\to\infty}\int_0^t \mathrm{d}t_1 \int_0^{t_1} \mathrm{d}t_2 \int_0^{t_2} \mathrm{d}t_3 e^{-i(\omega_1 t_1+\omega_2 t_2+\omega_3 t_3)} e^{i(\omega_1+\omega_2+\omega_3)t} c_i(t-t_3)c_j(t_1-t_2) \tag{26}
$$

$$
R_{ij}^{(4)}(\omega_1,\omega_2,\omega_3) = \mathrm{Re}\lim_{t\to\infty}\int_0^t \mathrm{d}t_1 \int_0^{t_1} \mathrm{d}t_2 \int_0^{t_2} \mathrm{d}t_3 e^{-i(\omega_1 t_1+\omega_2 t_2+\omega_3 t_3)} e^{i(\omega_1+\omega_2+\omega_3)t} c_i^*(t-t_3)c_j(t_1-t_2) \tag{27}
$$

As discussed above, we neglect the imaginary part of the rates and only consider the long-time limit. In the limit $t \to \infty$, all $R_{ij}^{(\xi)}$ can be expressed in terms of just two functions

$$\alpha(\omega_i, \omega_j; \omega_1, \omega_2, \omega_3) = \frac{\delta(\omega_2 + \omega_3 - \omega_i - \omega_j)}{(\omega_1 + \omega_j)(\omega_2 - \omega_i)} - \frac{\delta(\omega_1 + \omega_2 + \omega_3 - \omega_i)}{(\omega_1 + \omega_j)(\omega_1 + \omega_2 - \omega_i + \omega_j)} - \frac{\delta(\omega_3 - \omega_j)}{(\omega_2 - \omega_i)(\omega_1 + \omega_2 - \omega_i + \omega_j)} \tag{28}$$

and

$$\beta(\omega_i, \omega_j; \omega_1, \omega_2, \omega_3) = \frac{\delta(\omega_2 + \omega_3 - \omega_i - \omega_j)}{(\omega_1 + \omega_j)(\omega_2 - \omega_j)} - \frac{\delta(\omega_1 + \omega_2 + \omega_3 - \omega_i)}{(\omega_1 + \omega_j)(\omega_1 + \omega_2)} - \frac{\delta(\omega_3 - \omega_i)}{(\omega_2 - \omega_j)(\omega_1 + \omega_2)} \tag{29}$$

as

$$R_{ij}^{(1)}(\omega_1, \omega_2, \omega_3) = \pi \Big[+ \alpha(+\omega_i, +\omega_j; \omega_1, \omega_2, \omega_3)\ (\bar{n}_i + 1)(\bar{n}_j + 1) \tag{30}$$
$$+ \alpha(+\omega_i, -\omega_j; \omega_1, \omega_2, \omega_3)\ (\bar{n}_i + 1)\bar{n}_j$$
$$+ \alpha(-\omega_i, +\omega_j; \omega_1, \omega_2, \omega_3)\ \bar{n}_i(\bar{n}_j + 1)$$
$$+ \alpha(-\omega_i, -\omega_j; \omega_1, \omega_2, \omega_3)\ \bar{n}_i\bar{n}_j \Big]$$

$$R_{ij}^{(2)}(\omega_1, \omega_2, \omega_3) = \pi \Big[+ \alpha(-\omega_i, +\omega_j; \omega_1, \omega_2, \omega_3)\ (\bar{n}_i + 1)(\bar{n}_j + 1) \tag{31}$$
$$+ \alpha(-\omega_i, -\omega_j; \omega_1, \omega_2, \omega_3)\ (\bar{n}_i + 1)\bar{n}_j$$
$$+ \alpha(+\omega_i, +\omega_j; \omega_1, \omega_2, \omega_3)\ \bar{n}_i(\bar{n}_j + 1)$$
$$+ \alpha(+\omega_i, -\omega_j; \omega_1, \omega_2, \omega_3)\ \bar{n}_i\bar{n}_j \Big]$$

$$R_{ij}^{(3)}(\omega_1, \omega_2, \omega_3) = \pi \Big[+ \beta(+\omega_i, +\omega_j; \omega_1, \omega_2, \omega_3)\ (\bar{n}_i + 1)(\bar{n}_j + 1) \tag{32}$$
$$+ \beta(+\omega_i, -\omega_j; \omega_1, \omega_2, \omega_3)\ (\bar{n}_i + 1)\bar{n}_j$$
$$+ \beta(-\omega_i, +\omega_j; \omega_1, \omega_2, \omega_3)\ \bar{n}_i(\bar{n}_j + 1)$$
$$+ \beta(-\omega_i, -\omega_j; \omega_1, \omega_2, \omega_3)\ \bar{n}_i\bar{n}_j \Big]$$

$$R_{ij}^{(4)}(\omega_1, \omega_2, \omega_3) = \pi \Big[+ \beta(-\omega_i, +\omega_j; \omega_1, \omega_2, \omega_3)\ (\bar{n}_i + 1)(\bar{n}_j + 1) \tag{33}$$
$$+ \beta(-\omega_i, -\omega_j; \omega_1, \omega_2, \omega_3)\ (\bar{n}_i + 1)\bar{n}_j$$
$$+ \beta(+\omega_i, +\omega_j; \omega_1, \omega_2, \omega_3)\ \bar{n}_i(\bar{n}_j + 1)$$
$$+ \beta(+\omega_i, -\omega_j; \omega_1, \omega_2, \omega_3)\ \bar{n}_i\bar{n}_j \Big].$$

We note that the first Dirac delta in Equations (28) and (29) corresponds to a two-phonon selection rule, whereas the second and third terms represent selection rules involving only one mode, and therefore describe effective single-phonon processes. Note that, whenever the denominator of the two-phonon term vanishes, the effective single-phonon terms cancel out the divergence.

We can now calculate the Raman-I rate for a transition between initial and final states ψ_i

and ψ_f by taking the expectation value $\langle\psi_f|\,\mathcal{K}_4\rho\,|\psi_f\rangle$ and collecting all terms proportional to $\langle\psi_i|\,\rho\,|\psi_i\rangle$. Upon expanding all the nested commutators in Equation (23), the TCL4 generator takes the form of a sum of terms where four different spin-phonon coupling operators act on the spin density matrix, from the left, right, or any possible combination, i.e. terms of the form $\hat{V}\hat{V}\hat{V}\hat{V}\rho$, $\hat{V}\hat{V}\hat{V}\rho\hat{V}$, $\hat{V}\hat{V}\rho\hat{V}\hat{V}$, $\hat{V}\rho\hat{V}\hat{V}\hat{V}$, and $\rho\hat{V}\hat{V}\hat{V}\hat{V}$, and with all possible permutations of mode indices i and j. Grouping together all terms with the same dependence on the operators $\hat{V}_i$, we obtain

$$
\begin{aligned}
(\mathcal{K}_4^I)_{ff,ii} = -2\ \mathrm{Re}\ \sum_{ij}\sum_{ab}\Big\{ & V_j^{fa}V_i^{ab}V_j^{bi}V_i^{if}\ r_{ij}^{(1)}(E_i,E_f,E_a,E_b) \\
& + V_i^{fa}V_j^{ab}V_j^{bi}V_i^{if}\ r_{ij}^{(2)}(E_i,E_f,E_a,E_b) \\
& + V_j^{fa}V_j^{ab}V_i^{bi}V_i^{if}\ r_{ij}^{(3)}(E_i,E_f,E_a,E_b) \\
& + V_i^{fa}V_j^{ai}V_i^{ib}V_j^{bf}\ r_{ij}^{(4)}(E_i,E_f,E_a,E_b) \\
& + V_i^{fa}V_j^{ai}V_j^{ib}V_i^{bf}\ r_{ij}^{(5)}(E_i,E_f,E_a,E_b)\Big\},
\end{aligned} \tag{34}
$$

with

$$r_{ij}^{(1)} = R_{ij}^{(1)}(\omega_{af},\omega_{ba},\omega_{ib}) + R_{ji}^{(2)}(\omega_{ab},\omega_{bi},\omega_{if}) + R_{ji}^{(3)}(\omega_{fi},\omega_{ba},\omega_{ib}) + R_{ji}^{(4)}(\omega_{ab},\omega_{if},\omega_{bi}) \tag{35}$$

$$r_{ij}^{(2)} = -R_{ij}^{(1)}(\omega_{ba},\omega_{af},\omega_{ib}) - R_{ij}^{(3)}(\omega_{ba},\omega_{ib},\omega_{af}) \tag{36}$$

$$r_{ij}^{(3)} = R_{ij}^{(3)}(\omega_{af},\omega_{ba},\omega_{ib}) - R_{ji}^{(2)}(\omega_{bi},\omega_{ab},\omega_{if}) - R_{ji}^{(3)}(\omega_{fi},\omega_{ib},\omega_{ba}) - R_{ji}^{(4)}(\omega_{bi},\omega_{if},\omega_{ab}) \tag{37}$$

$$r_{ij}^{(4)} = -R_{ij}^{(2)}(\omega_{fb},\omega_{bi},\omega_{ia}) - R_{ij}^{(3)}(\omega_{ai},\omega_{bf},\omega_{ib}) - R_{ij}^{(4)}(\omega_{fb},\omega_{ia},\omega_{bi}) \tag{38}$$

$$r_{ij}^{(5)} = R_{ij}^{(2)}(\omega_{bi},\omega_{fb},\omega_{ia}) + R_{ij}^{(3)}(\omega_{bi},\omega_{ia},\omega_{af}) + R_{ij}^{(4)}(\omega_{bi},\omega_{ia},\omega_{fb}). \tag{39}$$

The first three lines in Equation (34) correspond to terms of the form $\hat{V}\hat{V}\hat{V}\rho\hat{V}$ and $\hat{V}\rho\hat{V}\hat{V}\hat{V}$, while lines four and five correspond to $\hat{V}\hat{V}\rho\hat{V}\hat{V}$, while there is no contribution from terms of the form $\rho\hat{V}\hat{V}\hat{V}\hat{V}$ or $\hat{V}\hat{V}\hat{V}\hat{V}\rho$. All five terms in Equation (34) can contribute to population dynamics via the effective single-phonon transitions arising from Equations (28) and (29),

and thus can be seen as higher order corrections to the Orbach process. However, it can be shown that only the last two lines contribute to genuine two-phonon Raman transitions. Focussing only on these terms, we can finally write the Raman-I rate as

$$
\begin{aligned}
(\mathcal{K}_4^I)_{ff,ii} = \pi \sum_{ij} \Big[& \eta^{fi}(+\omega_i, +\omega_j)\ \delta(\omega_{if} - \omega_i - \omega_j)\ (\bar{n}_i + 1)\ (\bar{n}_j + 1) \\
& + \eta^{fi}(+\omega_i, -\omega_j)\ \delta(\omega_{if} - \omega_i + \omega_j)\ (\bar{n}_i + 1)\ \bar{n}_j \\
& + \eta^{fi}(-\omega_i, +\omega_j)\ \delta(\omega_{if} + \omega_i - \omega_j)\ \bar{n}_i\ (\bar{n}_j + 1) \\
& + \eta^{fi}(-\omega_i, -\omega_j)\ \delta(\omega_{if} + \omega_i + \omega_j)\ \bar{n}_i\ \bar{n}_j \Big],
\end{aligned} \tag{40}
$$

with

$$
\eta^{fi}(\omega_i, \omega_j) = \left| \sum_a \left(\frac{V_i^{fa} V_j^{ai}}{\omega_{ai} + \omega_j} + \frac{V_j^{fa} V_i^{ai}}{\omega_{ai} + \omega_i} \right) \right|^2 . \tag{41}
$$

References

(1) Kresse, G.; Hafner, J. *Ab initio* molecular dynamics for liquid metals. *Phys. Rev. B* **1993**, *47*, 558–561.

(2) Togo, A.; Tanaka, I. First principles phonon calculations in materials science. *Scripta Materialia* **2015**, *108*, 1–5.

(3) Perdew, J. P.; Burke, K.; Ernzerhof, M. Generalized Gradient Approximation Made Simple. *Phys. Rev. Lett.* **1996**, *77*, 3865–3868.

(4) Grimme, S.; Antony, J.; Ehrlich, S.; Krieg, H. A consistent and accurate ab initio parametrization of density functional dispersion correction (DFT-D) for the 94 elements H-Pu. *J. Chem. Phys.* **2010**, *132*, 154104.

(5) Blöchl, P. E. Projector augmented-wave method. *Phys. Rev. B* **1994**, *50*, 17953–17979.

(6) Kresse, G.; Joubert, D. From ultrasoft pseudopotentials to the projector augmented-wave method. *Phys. Rev. B* **1999**, *59*, 1758–1775.

(7) Perdew, J. P.; Ruzsinszky, A.; Csonka, G. I.; Vydrov, O. A.; Scuseria, G. E.; Constantin, L. A.; Zhou, X.; Burke, K. Restoring the Density-Gradient Expansion for Exchange in Solids and Surfaces. *Phys. Rev. Lett.* **2008**, *100*, 136406.

(8) Monkhorst, H. J.; Pack, J. D. Special points for Brillouin-zone integrations. *Phys. Rev. B* **1976**, *13*, 5188–5192.

(9) Gonze, X.; Charlier, J.-C.; Allan, D.; Teter, M. Interatomic force constants from first principles: The case of α-quartz. *Phys. Rev. B* **1994**, *50*, 13035–13038.

(10) Gonze, X.; Lee, C. Dynamical matrices, Born effective charges, dielectric permittivity tensors, and interatomic force constants from density-functional perturbation theory. *Phys. Rev. B* **1997**, *55*, 10355–10368.

(11) Gajdoš, M.; Hummer, K.; Kresse, G.; Furthmüller, J.; Bechstedt, F. Linear optical properties in the projector-augmented wave methodology. *Phys. Rev. B* **2006**, *73*, 045112.

(12) Jain, A.; Ong, S. P.; Hautier, G.; Chen, W.; Richards, W. D.; Dacek, S.; Cholia, S.; Gunter, D.; Skinner, D.; Ceder, G.; Persson, K. A. The Materials Project: A materials genome approach to accelerating materials innovation. *APL Materials* **2013**, *1*, 011002.

(13) Skelton, J. M. `MolecularCrystalPhononAnimation`. `https://github.com/JMSkelton/MolecularCrystalPhononAnimation`.

(14) Breuer, H.-P.; Petruccione, F.; Petruccione, *The Theory of Open Quantum Systems*; Oxford University Press, 2002.
